# Targeting HIV-1 Env gp140 to LOX-1 Elicits Immune Responses in Rhesus Macaques

**DOI:** 10.1371/journal.pone.0153484

**Published:** 2016-04-14

**Authors:** Gerard Zurawski, Sandra Zurawski, Anne-Laure Flamar, Laura Richert, Ralf Wagner, Georgia D. Tomaras, David C. Montefiori, Mario Roederer, Guido Ferrari, Christine Lacabaratz, Henri Bonnabau, Peter Klucar, Zhiqing Wang, Kathryn E. Foulds, Shing-Fen Kao, Nicole L. Yates, Celia LaBranche, Bertram L. Jacobs, Karen Kibler, Benedikt Asbach, Alexander Kliche, Andres Salazar, Steve Reed, Steve Self, Raphael Gottardo, Lindsey Galmin, Deborah Weiss, Anthony Cristillo, Rodolphe Thiebaut, Giuseppe Pantaleo, Yves Levy

**Affiliations:** 1 Vaccine Research Institute, Université Paris-Est, Faculté de Médecine, INSERM U955, and Assistance Publique-Hôpitaux de Paris, Groupe Henri-Mondor Albert- Chenevier, service d’immunologie clinique, INRIA SISTM, Créteil, France; 2 Baylor Institute for Immunology Research and INSERM U955, Dallas, Texas, United States of America; 3 INSERM U897, INRIA SISTM, Université Bordeaux Segalen, Bordeaux, France; 4 Molecular Microbiology and Gene Therapy Unit, Institute of Medical Microbiology and Hygiene, University of Regensburg, Regensburg, Germany; 5 Department of Surgery, Duke University Medical Center, Durham, North Carolina, United States of America; 6 Duke Human Vaccine Institute, Duke University Medical Center, Durham, North Carolina, United States of America; 7 Vaccine Research Center, NIAID, NIH, Bethesda, Maryland, United States of America; 8 School of Life Sciences, Center for Infectious Diseases and Vaccinology, Arizona State University, Tempe, Arizona, United States of America; 9 Vaccine and Infectious Disease and Public Health Sciences Divisions, Fred Hutchinson Cancer Research Center, Seattle, Washington, United States of America; 10 Oncovir, Washington, D.C., United States of America; 11 Infectious Disease Research Institute, Seattle, Washington, United States of America; 12 Advanced BioScience Laboratories, Inc., Rockville, Maryland, United States of America; 13 Centre Hospitalier Universitaire Vaudois, CH-101, Lausanne, Switzerland; University of Massachusetts Medical School, UNITED STATES

## Abstract

Improved antigenicity against HIV-1 envelope (Env) protein is needed to elicit vaccine-induced protective immunity in humans. Here we describe the first tests in non-human primates (NHPs) of Env gp140 protein fused to a humanized anti-LOX-1 recombinant antibody for delivering Env directly to LOX-1-bearing antigen presenting cells, especially dendritic cells (DC). LOX-1, or 1ectin-like oxidized low-density lipoprotein (LDL) receptor-1, is expressed on various antigen presenting cells and endothelial cells, and is involved in promoting humoral immune responses. The anti-LOX-1 Env gp140 fusion protein was tested for priming immune responses and boosting responses in animals primed with replication competent NYVAC-KC Env gp140 vaccinia virus. Anti-LOX-1 Env gp140 vaccination elicited robust cellular and humoral responses when used for either priming or boosting immunity. Co-administration with Poly ICLC, a TLR3 agonist, was superior to GLA, a TLR4 agonist. Both CD4^+^ and CD8^+^ Env-specific T cell responses were elicited by anti-LOX-1 Env gp140, but in particular the CD4^+^ T cells were multifunctional and directed to multiple epitopes. Serum IgG and IgA antibody responses induced by anti-LOX-1 Env gp140 against various gp140 domains were cross-reactive across HIV-1 clades; however, the sera neutralized only HIV-1 bearing sequences most similar to the clade C 96ZM651 Env gp140 carried by the anti-LOX-1 vehicle. These data, as well as the safety of this protein vaccine, justify further exploration of this DC-targeting vaccine approach for protective immunity against HIV-1.

## Introduction

The RV144 Thai HIV vaccine trial gave some hope that protective immunity could be evoked by a combination of priming via a viral vector with HIV Env protein plus adjuvant as a boost. However, vaccine efficacy was modest at 60% at year 1 and waned rapidly [1,2]. Thus, current preventative HIV vaccine development efforts are focused on optimizing such priming and boosting components and investigating vaccination regimens. An approach to increasing protein antigen efficacy is their selective delivery to endocytic receptors on dendritic cell (DC) surfaces, the key cell type for initiating and regulating immune responses [3]. Indeed, immunization of nonhuman primates (NHPs) with an anti-DEC-205 antibody fused to HIV Gag p24 followed by a boost with recombinant New York vaccinia (NYVAC) virus bearing p24 induced robust T cell and humoral immunity [4]. However, antibody responses directed to the HIV-1 coat protein Env are key to protective humoral responses. To this end, we have developed as a candidate protein a DC-targeting vaccine bearing gp140 from clade C 96ZM651 fused to a humanized anti-human LOX-1 recombinant IgG4 antibody, termed αLOX-1.Env gp140. LOX-1, or lectin-like oxidized low density lipoprotein receptor, is a C-type lectin pattern recognition receptor, and *in vitro* and *in vivo* studies show that targeting antigens to LOX-1 instructs DCs and B cells to promote the generation of mucosal plasmablast differentiation as well as eliciting CD4^+^ T cell responses with a Th1 phenotype [5,6], thus making it an attractive candidate endocytic receptor for targeting Env protein.

Our study tests the relative efficacy of antibody and T cell responses directed to Env gp140 in NHPs vaccinated with αLOX-1.HIV Env gp140 co-administered with either poly ICLC (Toll-like receptor 3 or TLR3 agonist) or Glucopyranosyl Lipid Adjuvant (GLA, TLR4 agonist) as adjuvants and vaccine assessment was conducted in animals either primed or further boosted with a replication-competent viral vector (NYVAC-KC) bearing Env gp140 and GagPolNef. We found that the αLOX-1.Env gp140 fusion protein elicited robust anti-Env serum antibody responses, either as a prime or in boosting viral-based vaccination. The combination of αLOX-1.Env gp140 with poly ICLC was particularly favorable for both antibody and T cell responses to Env gp140.

## Results

### Characteristics of anti-LOX-1 antibody fused to Env gp140 protein

**Fig 1:**
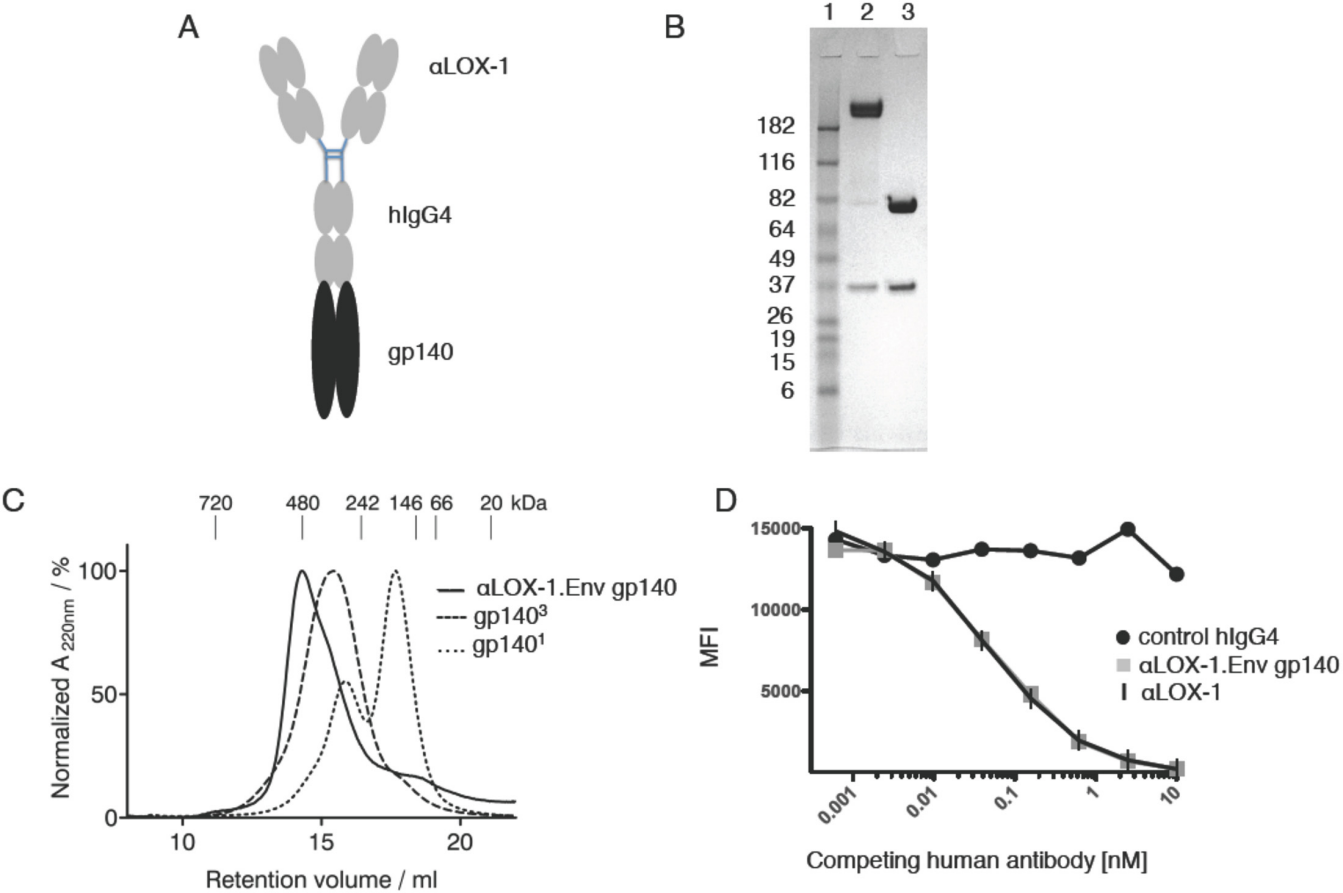
Design and physical properties of αLOX-1.Env gp140 fusion protein. (A) Schematic representation showing antibody (grey) fused at the H chain C-terminus to HIV Env gp140 (black). (B) Analysis of 3 μg purified αLOX-1.Env gp140 fusion protein (lane 2) or 3 μg purified αLOX-1 protein (lane 3) by reducing SDS-PAGE stained with Coomassie Blue and location of protein molecular weight markers (lane 1) is indicated in kDa. Unglycosylated mass of αLOX-1 L chain is ca. 23,800 Da and of αLOX-1.Env gp140 H chain is ca. 127,570 Da. (C) Size exclusion gel chromatography analysis of αLOX-1.Env gp140. The proteins were run separately on an TSK G4000SW column in PBS at 0.5 ml/min. αLOX-1.Env gp140 is shown in the solid line, trimeric gp140 is shown in the dashed line, and monomeric gp140 is shown in the dotted line. For molecular weight calibration the NativeMark Protein Standard (Life Technologies, LC0725) was used and their peak positions are indicated. (D) Equilibrium competition binding analysis of αLOX-1.HIV Env gp140 interaction with human LOX-1. Beads coated with human LOX-1 ectodomain were incubated overnight with 10 ng/ml of the parental mouse αLOX-1 mAb and varying concentrations of αLOX-1.Env gp140 or humanized αLOX-1 IgG4 without fused antigen (X axis units are nM competing human αLOX-1 proteins), then probed with PE-labeled anti-mouse IgG, and analyzed by flow cytometry (Y axis units are mean fluorescence intensity). Black circles are an irrelevant control human IgG4 mAb, grey squares are human αLOX-1.Env gp140, and vertical strokes are humanized αLOX-1 without fused antigen.

We humanized the mouse variable regions of the previously described anti-human LOX-1 (αLOX-1) recombinant human IgG4 antibody vehicle that elicited protective humoral immunity in NHPs against Influenza virus when fused to influenza haemagglutinin HA1 [5,6]. Clade C 96ZM651 Env gp140 sequence was fused to the heavy (H) chain C-terminal codon and αLOX-1.Env gp140 protein was purified by protein A affinity chromatography from CHO-S cells stably transfected with vectors encoding αLOX-1.Env gp140 H chain and αLOX-1 L chain (Fig 1A). The product was heavily glycosylated based on reducing SDS-PAGE analysis (Fig 1B). Analysis of the protein complex by size exclusion chromatography showed that αLOX-1.Env gp140 forms a homogenous species with a peak size of ~500 kDa, which is consistent with the expected configuration of one antibody (ca. 150 kDa) plus two gp140 molecules. Thus, the dimerization of the antibody is dominant over the trimerization capacity of Env, and no higher-order complexes (such as minimally three antibody-dimers plus two gp140 trimers) are formed (Fig 1A–1C). αLOX-1.Env gp140 bound to human LOX-1 ectodomain protein equivalently compared to the parental recombinant antibody without fused antigen (Fig 1D) and retained cross-reactivity to NHP LOX-1 [5] (and not shown). Importantly, αLOX-1.Env gp140 maintained reactivity against a panel of neutralizing anti-Env antibodies that recognize distinct epitopes, although these interactions varied via both increased and decreased affinity compared to control trimeric 96ZM651 Env gp140 protein (S1 Table), suggesting minor distortions rather than abrogation of natural gp140 epitopes.

### αLOX-1.Env gp140 elicits Env-specific antibody responses in NHPs

The overall study design is shown in Table 1. In groups 1 and 2 (G1, G2), naïve Rhesus macaques were primed by two intramuscular (i.m.) injections, one month apart, containing 10^8^ pfu of separate replication competent NYVAC-KC viruses encoding: i) clade C 96ZM651 Gag with C/B’ clade 97CN54 PolNef, and ii) clade C 96ZM651 Env gp140 sequences [7]. Two months later, the NHPs were immunized three times monthly with 250 μg αLOX-1.Env gp140 injected intradermally (i.d.) in 8 sites around a single subcutaneous (s.c.) site administered with either Poly ICLC (G1; Hiltonol, 1 mg), a formulated TLR3 agonist, or GLA (G2; GLA, 20 μg), a synthetic TLR4 agonist. Ten weeks later, the NHPs in all groups were boosted with an additional i.m. injection of the NYVAC-KC viruses.

**Table 1:** Study design for testing immunogenicity of αLOX-1.Env gp140 in NHP.

	Group	Size	Week 0	Week 4	Week 12	Week 16	Week 20	Week 30
**1**	**Nkc2Lp3Nkc**	**6**	**NYVAC-KC**	**NYVAC-KC**	**αLOX-1.gp140 ⁄ poly ICLC**	**αLOX-1.gp140 ⁄ poly ICLC**	**αLOX-1.gp140 ⁄ poly ICLC**	**NYVAC-KC**
**2**	**Nkc2Lg3Nkc**	**6**	**NYVAC-KC**	**NYVAC-KC**	**αLOX-1.gp140 ⁄ GLA**	**αLOX-1.gp140 ⁄ GLA**	**αLOX-1.gp140 ⁄ GLA**	**NYVAC-KC**
**3**	**Lp3Nkc**	**4**	**Placebo**	**Placebo**	**αLOX-1.gp140 ⁄ poly ICLC**	**αLOX-1.gp140 ⁄ poly ICLC**	**αLOX-1.gp140 ⁄ poly ICLC**	**NYVAC-KC**
**4**	**Lg3Nkc**	**4**	**Placebo**	**Placebo**	**αLOX-1.gp140 ⁄ GLA**	**αLOX-1.gp140 ⁄ GLA**	**αLOX-1.gp140 ⁄ GLA**	**NYVAC-KC**

The table shows the immunization regimens used in this study. NYVAC-KC is a mixture of replication competent viruses encoding: i) HIV B/C clade CN54 PolNef with clade C 96ZM651 Gag, and ii) clade C 96ZM651 Env gp140 sequences. Poly ICLC and GLA are co-administered adjuvants. Week refers to sample times calculated from initiation of the study (Week 0). For clarity, the groups are referred to as G1 Nkc2Lp3Nkc, G2 Nkc2Lg3Nkc, G3 Lp3Nkc, and G4 Lg3Nkc.

**Fig 2:**
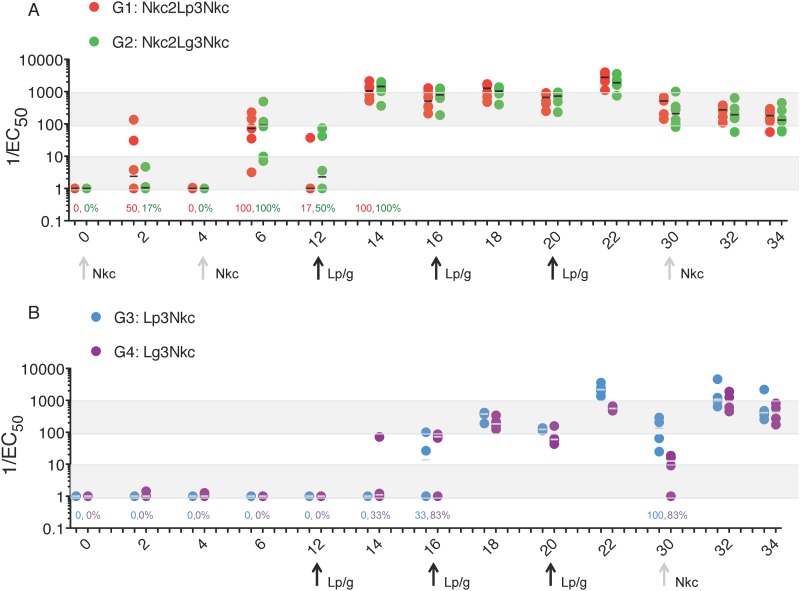
Serum Env gp140-specific IgG responses to αLOX-1.Env gp140 administration. (A) Responses in NHP (n = 6 per group) primed with NYVAC-KC virus at week 0 and week 4 (X axis values), followed by αLOX-1.Env gp140 administrations at weeks 12, 16, and 20, with an additional NYVAC-KC boost at week 30 are shown. (B) Responses in NHP primed with αLOX-1.Env gp140 administrations at weeks 12, 16, and 20 (n = 4 per group), with a NYVAC-KC virus boost at week 30 are shown. Open symbols are with poly ICLC co-administration and closed symbols are with GLA co-administration. Reciprocal of the half maximal titration by solid-phase ELISA versus Env gp140 protein is plotted for individual NHPs. Bars are the median. Response rates, based on 1/EC_50_ >2, are shown above the X-axis and are 100% unless otherwise indicated.

Serum Env-specific IgG levels from G1 Nkc2Lp3Nkc and G2 Nkc2Lg3Nkc were undetectable or very low after the NYVAC-KC injections through to the first αLOX-1.Env gp140 administration at week 12 (Fig 2A). However at week 14, two weeks after the first αLOX-1.Env gp140 injection, serum Env-specific IgG titers were readily detected in all NHPs. The two subsequent injections of αLOX-1.Env gp140 further boosted titers, which had waned somewhat from the peaks at 2 weeks after each injection. Maximal titers were achieved at week 22, two weeks after the third αLOX-1.Env gp140 injection (P = 0.016 for both comparison to baseline; Wilcoxon signed-rank tests), and there was no significant difference between αLOX-1.Env gp140 adjuvanted with poly ICLC versus GLA (tested Relative to a Random Effect Model, P = 0.7). Eight weeks after the observed peak at week 22, titers again decreased and were not boosted by the additional NYVAC-KC administration at week 30. However, the magnitude of IgG responses remained higher than baseline values (P = 0.016 for both groups).

NHP groups G3 Lp3Nkc and G4 Lg3Nkc received placebo instead of NYVAC-KC at weeks 0 and 4 (Table 1). Undetectable or very low serum anti-Env titers were present until two weeks after the second αLOX-1.Env gp140 administration (week 16), but were boosted to maximal values two weeks after the third αLOX-1.Env gp140 administration (week 22) (P = 0.06 in both groups as compared to baseline values), especially in G3 with poly ICLC as adjuvant (Fig 2A). Week 22 peak titers waned through to week 30, but were boosted by a single NYVAC-KC injection at week 30 in both groups. For G3 versus G4, there was a significant advantage for the poly ICLC adjuvant versus GLA adjuvant at week 22, but there was no difference in the boost from the NYVAC-KC administration at weeks 32 and 34 (respectively, P = 0.02, P = 0.68, and P = 0.88; Wilcoxon Rank Sum Test).

At weeks 22 and 32 sera from all four NHP groups were tested for Env-specific IgG titers against Env gp120 and gp41 subdomains. All groups raised serum titers specific to both the gp120 and gp41subdomains of Env gp140 (S1 Fig).

**Fig 3:**
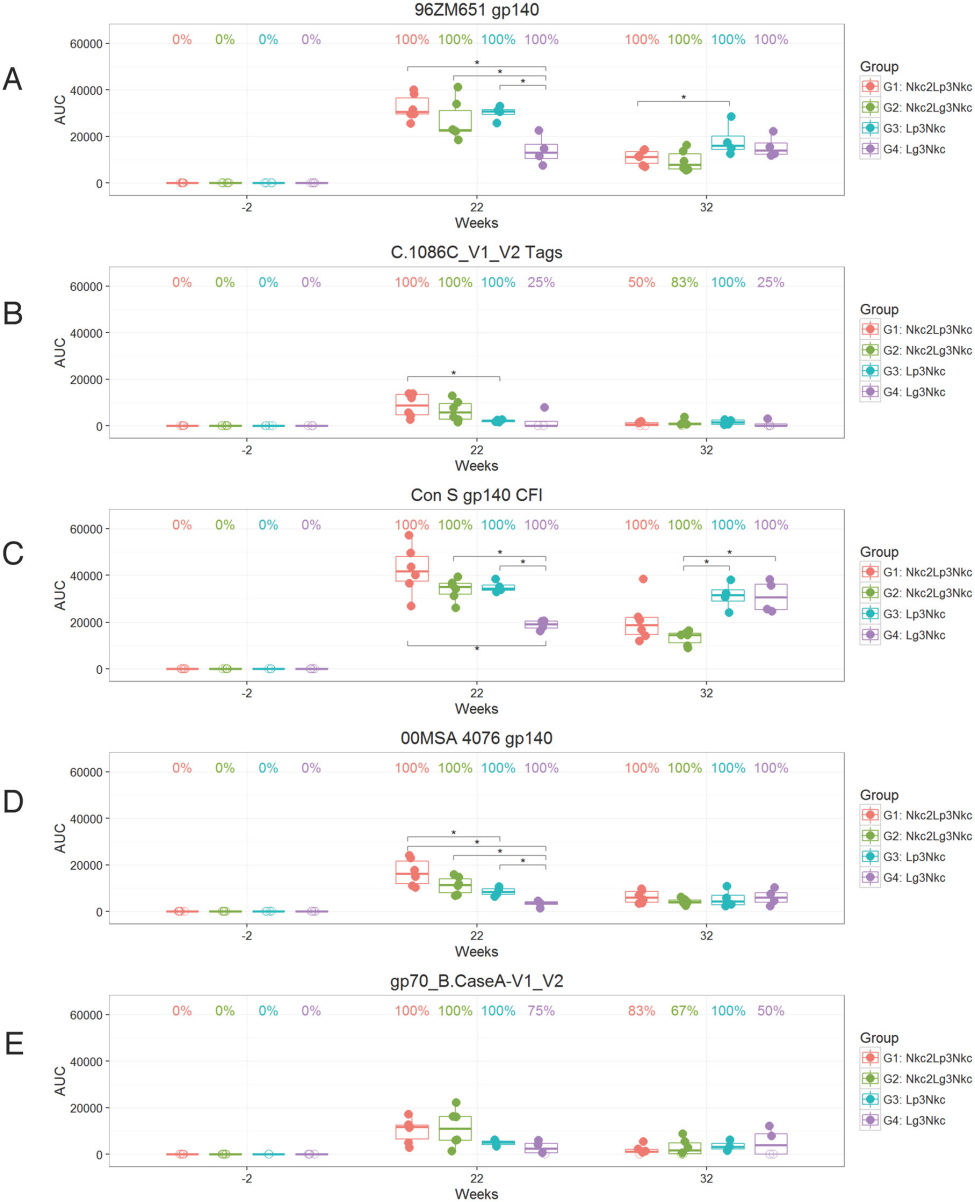
Analysis of Env binding serum IgG antibody titers. Plasma samples taken at week -2 (baseline), week 22 (2 weeks post αLOX-1.Env gp140 vaccinations), and week 32 (2 weeks post NYVAC-KC boost) were measured by binding antibody multiplex assay against the indicated Env proteins: (A) 96ZM651 gp140, (B) C.1086C_V1_V2 Tags, (C) Con S gp140 CFI, (D) 00MSA 4076, and (E) gp70_B.CaseA-V1_V2. Each panel shows anti-Env IgG titers represented as an area under the titration curve (AUC) of individual NHP (n = 6 for G1, G2; n = 4 for G3, G4). Boxplots show the 25th, 50th, and 75th percentiles of the AUC distribution for each group. Filled and open circles denote positive and negative responders, respectively, and the percent of positive responders is noted above each boxplot. AUC comparisons between groups where the Wilcoxon rank sum test p-value is < 0.05 are denoted with a *. Note that part of the data (weeks -2, 22, 32) in (A) is replicated independently in Fig 2.

Sera from all four NHP groups were also tested for Env-specific IgG titers against a wider array of HIV-1 Env antigens including clade C.1086V1-V2 Tags, Con S gp140 CFI (a group M consensus envelope gp140), clade A 00MSA 4076 gp140 (Fig 3A–3D), and A1.con.env03 140 CF (clade A Consensus), B.con.env03 140 CF (clade B Consensus), C.con.env03 140 CF (clade C Consensus), JRFL gp140 (clade B), gp70 control, and gp70_B.CaseA2 V1/V2 (not shown). Sera from the week 22 and week 32 samples gave positive IgG response rates to all the Env proteins to those observed against the clade C 96ZM651 Env gp140 protein that was vaccinated sequence (Fig 3A). At week 22, cross-clade IgG binding antibody responses were detected in all NHPs (Fig 3A–3D), with G1 Nkc2Lp3Nkc, G2 NkcLg3Nkc, and G3 Lp3Nkc having significantly higher responses than G4 Lg3Nkc for most envelope proteins. G1 Nkc2Lp3Nkc IgG responses generally trended higher than G3 Lp3Nkc, with differences noted in the clade A gp140 protein (00MSA 4076 gp140) and clade C V1-V2 antigen (C.1086C_V1_V2 Tags) (P<0.05, Wilcoxon Rank Sum Test). However, at week 32, IgG binding titers (AUC) trended higher in G3 and G4 Lg3Nkc as compared to G1 and G2 Nkc2Lp3Nkc with significant differences noted for the vaccine strains gp140 (96ZM651 gp140) and Con S gp140 CFI (P<0.05, Wilcoxon Rank Sum Test). There were no significant differences in response rates between groups for any antigen at week 22 or 32. Thus, the magnitude of the envelope antibody response is improved after the last NYVAC-KC immunization in the absence of NYVAC-KC prime. At week 22, IgG binding antibody titers to most envelope proteins trended higher in G1 Nkc2Lp3Nkc compared to G2 Nkc2Lg3Nkc (Fig 3A–3D) with the clade B gp140 proteins (B.con.env03; JRFL gp140) scoring significantly higher for G1 than G3 (P<0.05, Wilcoxon Rank Sum Test, not shown).

**Fig 4:**
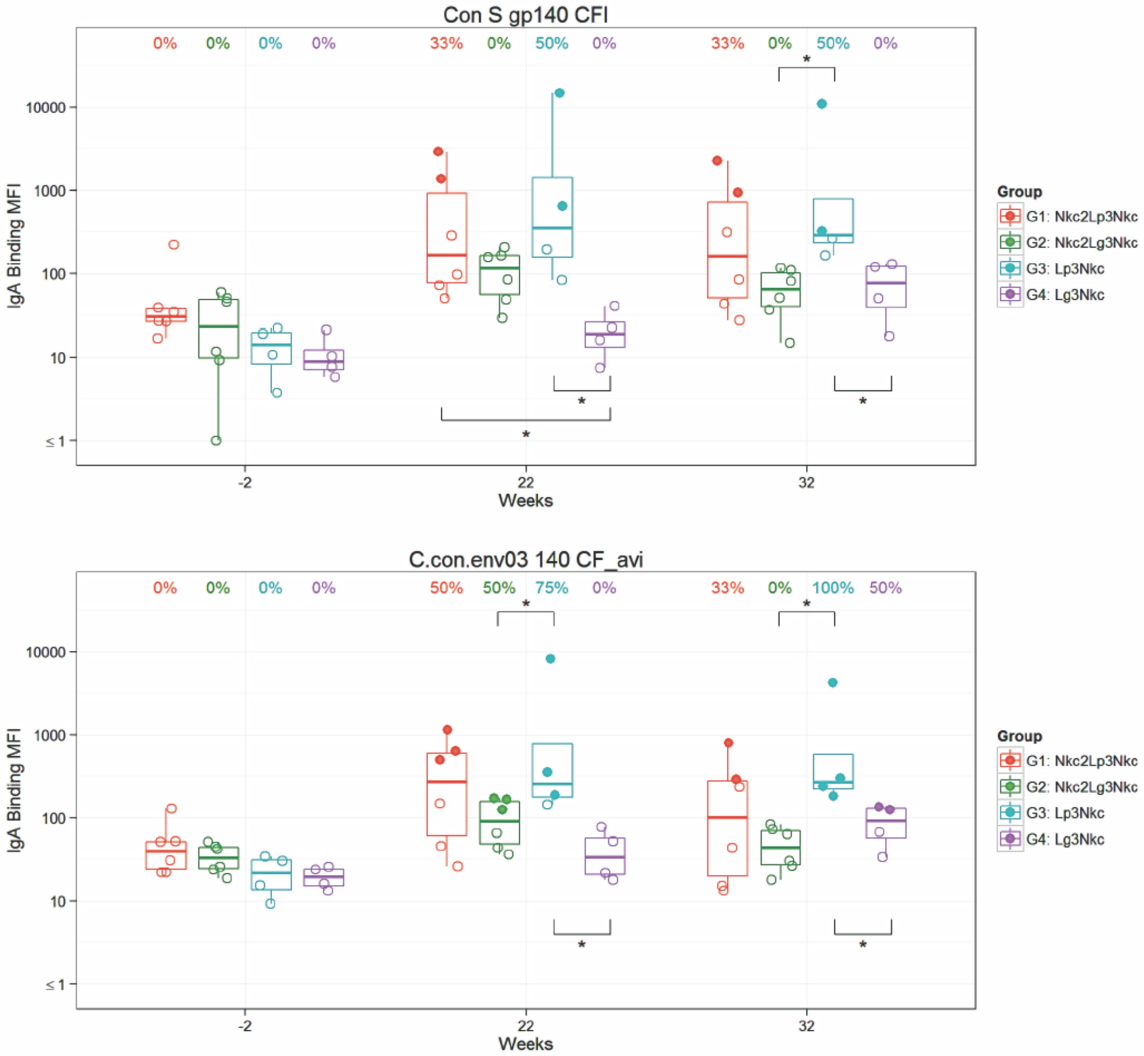
Analysis of Env-specific IgA binding levels. Plasma samples taken at week -2 (baseline), week 22 (2 weeks post αLOX-1.Env gp140 administrations), and week 32 (2 weeks post NYVAC-KC boost) were measured by binding antibody multiplex assay against the Env proteins indicated above each panel. Each panel shows anti-Env IgA binding units (MFI) of individual NHPs NHP (n = 6 for G1, G2; n = 4 for G3, G4). Plotting details are as described in Fig 3, except IgA levels were log_10_-transformed. Non-responders (open circles) did not meet the pre-specified criteria for vaccine-induced positivity as outlined in the Methods.

Globally, serum IgA responses were lower compared to IgG binding responses (Fig 4). Binding levels were higher in the poly ICLC adjuvant groups G1 Nkc2Lp3Nkc and G3 Lp3Nkc and there were no significant differences between these groups at week 22 or week 32 in response rates and titers.

**Fig 5:**
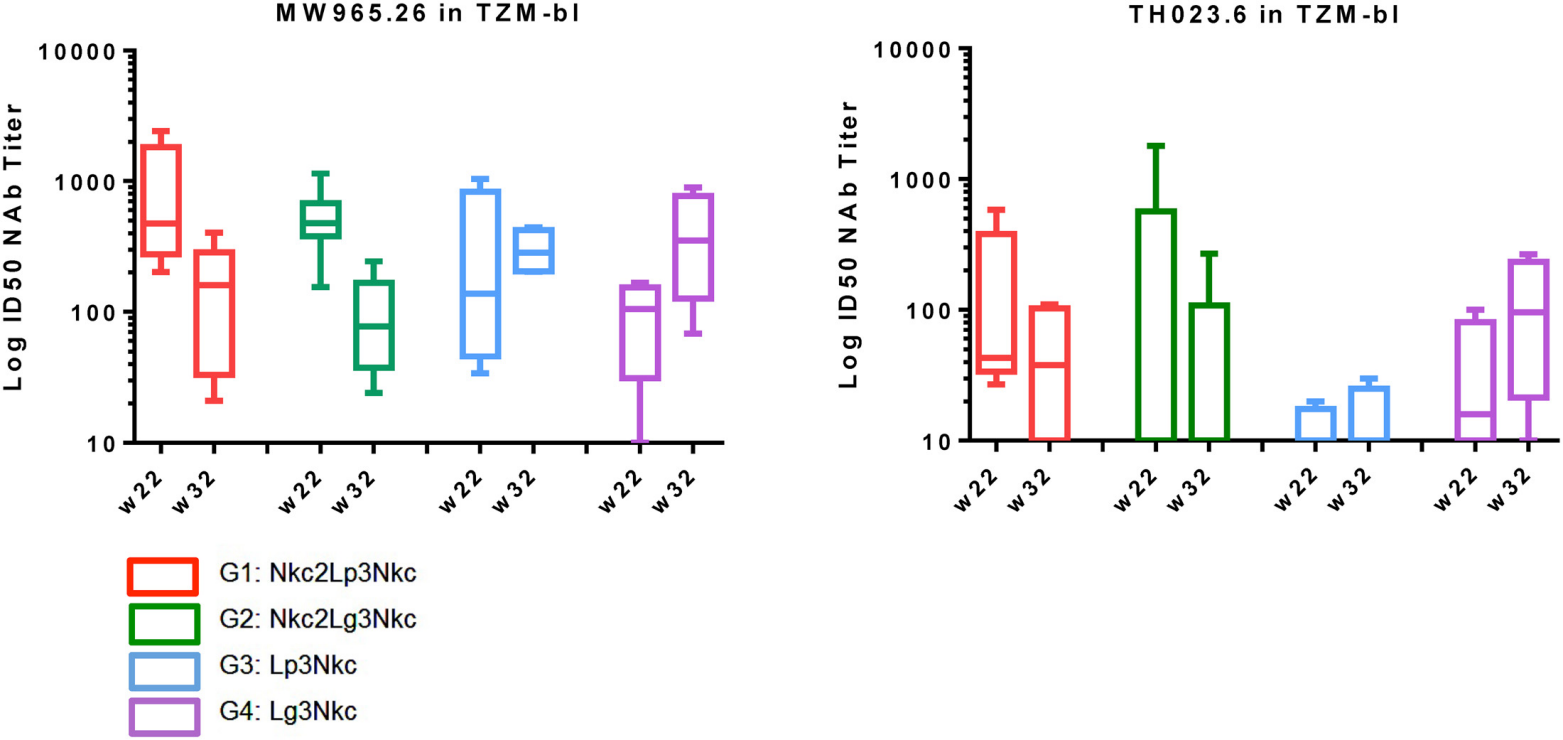
Analysis of neutralizing antibody titers. Plasma samples taken at peak response week 22 (2 weeks post αLOX-1.Env gp140 administrations) and week 32 (2 weeks post NYVAC-KC boost) for groups 1–4 (G1-G4) were tested for neutralizing titers versus each indicated virus (MW965.26 or TH023.6). Log to base 10 of titers inhibiting replication by 50% for individual NHP (n = 6 for G1, G2; n = 4 for G3, G4) are shown. Boxes represent the middle 95^th^ percentile. Horizontal lines are the median. S2 and S3 Tables show similar tests against other viruses.

Analysis of HIV-1 neutralizing activity in week 22 and week 32 plasma samples was performed via TZM.bl assays against a panel of Tier 1 and Tier 2 HIV-1 Env pseudotyped viruses. Neutralizing activity was detected against MW965.26 (clade C, Tier 1A, Fig 5) and TH023.6 (CRF01_AE, Tier 1A, Fig 5). Little or no neutralization was detected against viruses MN.3 and SF162.LS (clade B, Tier 1A), Bal.26 (clade B, Tier 1B) (S2 Table). Also, no neutralization was detected against the clade C Tier 2 viruses TV1.21, Ce1086_B2 and 96ZM651 (S2 Table). Neutralization titers against the sensitive viruses (MW965.26 and TH023.6) decreased from week 22 to week 32 in groups G1 Nkc2Lp3Nkc and G2 Nkc2Lg3Nkc, but was similar or increased in groups G3 Lp3Nkc and G4 Lg3Nkc.

ADCC-mediated antibody responses were measured at week 0, 6, 22, and 32, but were uninformative due to unexplained high response rates at baseline (S2 Fig).

### αLOX-1.Env gp140 elicits Env-specific T cell responses in NHP

**Fig 6:**
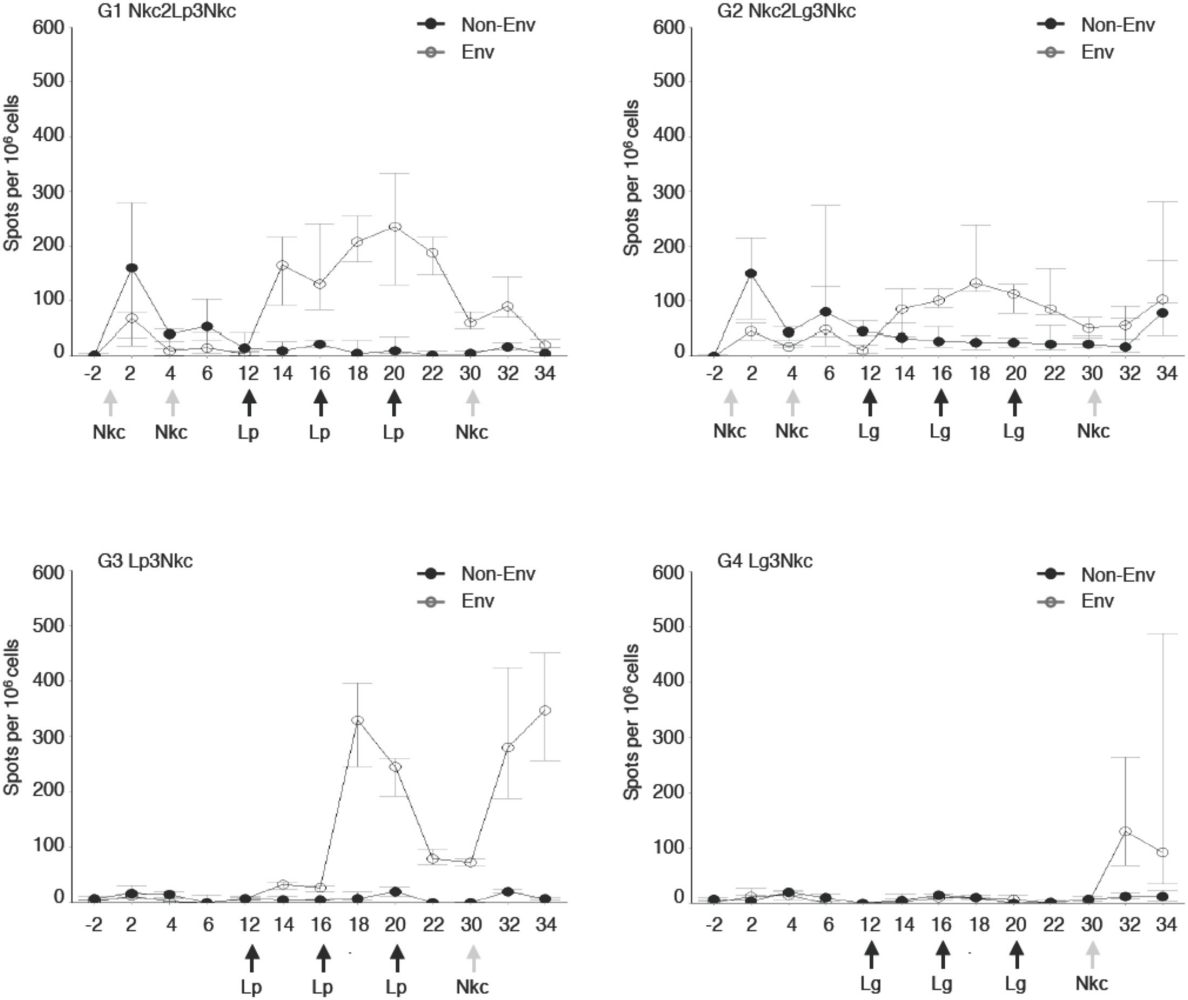
Analysis by IFNγ ELISPOT of blood T cell responses. Reponses in NHP in the indicated NHP groups (n = 6 for G1, G2; n = 4 for G3, G4) are shown averaged for each group for each sampling time (shown in the X axis as weeks relative to initiation of the vaccinations). Filled points are the sum of T cell responses specific to 6 peptide pools for Gag, Pol, and Nef (i.e., responses to NYVAC-KC Gag Nef Pol), while open points are summed averages of T cell responses specific to Env gp140 peptide pools (i.e., response to NYVAC-KC Env gp140 and/or αLOX-1.Env gp140). Medians for each group at each sampling time are presented and bars are the quartile 25–75 range. Arrows below the X-axis indicate the time points for NYVAC-KC (Nkc) and the DC-targeting vaccinations with poly ICLC (Lp) or GLA (Lg).

T cell responses specific to the non-Env antigens delivered by the NYVAC-KC bearing Gag, Pol, and Nef, as well as Env-specific T cell responses from either the NYVAC-KC bearing Env gp140 and the αLOX-1.Env gp140 vaccine, were monitored by IFNγ ELISPOT analysis of PBMCs. In NHP groups G1 Nkc2Lp3Nkc and G2 Nkc2Lg3Nkc, NYVAC-KC elicited significant T cell responses against non-Env antigens (P = 0.036 for both groups at week 2 as compared to baseline, Wilcoxon signed-rank tests), whereas no significant increase of responses against Env was noted at two weeks post the second NYVAC-KC administration (Fig 6). At week 14, 2 weeks after the first αLOX-1.Env gp140 boost, Env-specific T cells were increased significantly (P = 0.031 as compared to baseline), while non-Env-specific T cell numbers waned compared to their peak 2 weeks after the second virus injection. In contrast to the antibody responses, blood Env-specific T cell levels were maintained during the αLOX-1.Env gp140 vaccinations, subsequently dropped by ~2-fold, and were not markedly boosted with the additional NYVAC-KC injection. However, responses against Env and non-Env peptides remained significantly higher than baseline values in both groups (P = 0.03). Analysis of responses defined by separate peptide pools (not shown) indicated that both non-Env and Env-specific T cell responses were directed to multiple epitopes. There were no significant differences between G1 Nkc2Lp3Nkc and G2 Nkc2Lg3Nkc in the overall magnitude or epitope spread for the T cell responses, although the responses trended higher in the poly ICLC adjuvanted G1.

In G3 Lp3Nkc, αLOX-1.Env gp140 co-administered with poly ICLC elicited detectable circulating Env-specific T cell at 2 and 4 weeks after the first administration, the second dose raised these levels several-fold (Fig 6). After the second vaccination, the levels waned and stabilized through week 30, but were restored to peak levels by the NYVAC-KC boost. In contrast, in the GLA adjuvanted G4 Lg3Nkc, αLOX-1.Env gp140 failed to elicit detectable circulating Env-specific T cells. The single virus administration at week 30 elicited moderate Env-specific T cell counts similar to those seen in groups G1 and G3 two weeks after a single NYVAC-KC administration (Fig 6). Analysis of responses defined by separate Env peptide pools (not shown) indicated that Env-specific T cell responses were directed to multiple epitopes and there were no apparent differences between G3 Lp3Nkc and G4 Lg3Nkc in the T cell epitope regions of the response to Env.

**Fig 7:**
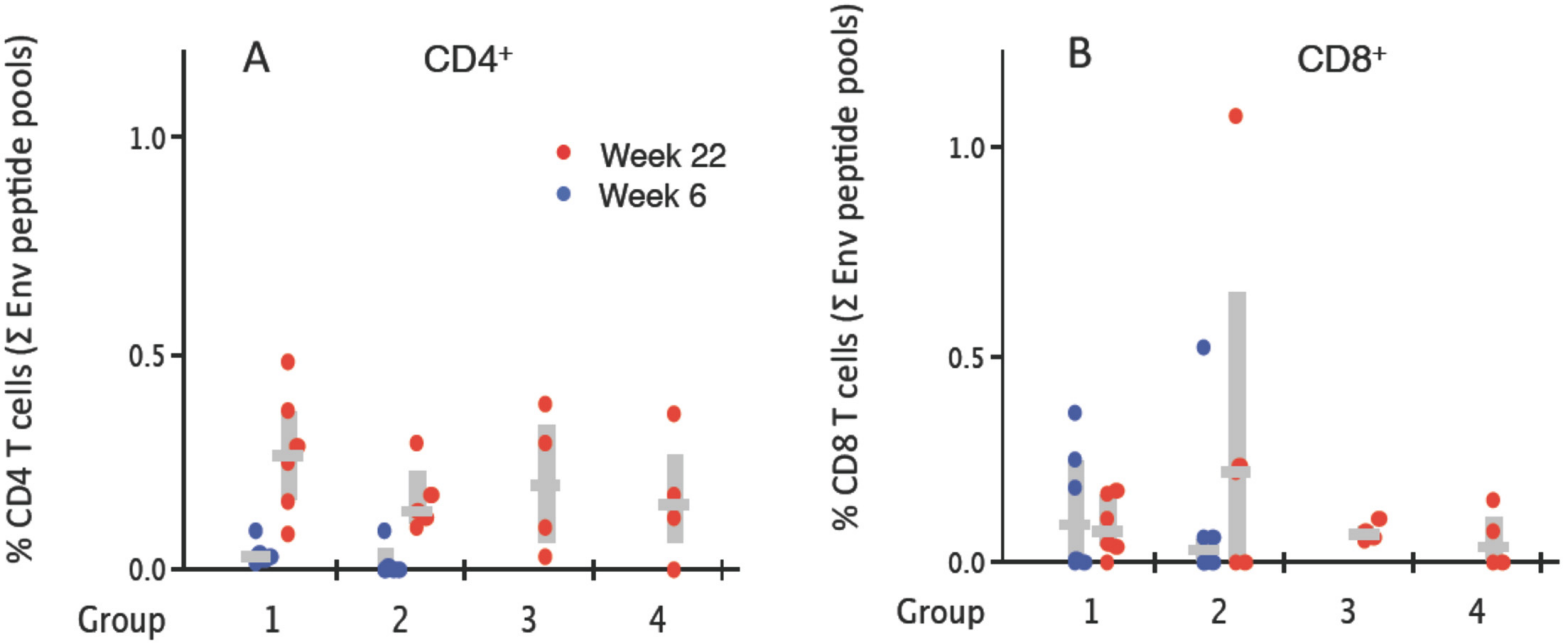
Analysis of HIV Env-specific cytokine-producing T cells. (A) Env peptide-specific CD4^+^ T cell responses and (B) Env peptide-specific CD8^+^ T cell responses from PBMC samples taken at week 6 (2 weeks post NYVAC-KC administration for G1 and G2 only) (blue circles) and week 22 (2 weeks post the final αLOX-1.Env gp140 administration) (red circles). The data are based on total CD4^+^ and CD8^+^ T cells and show the sum of IFNγ^+^, IL-2^+^, and TNFα^+^ T cells specific to the Env peptide pools for individual NHP as filled circles. The grey horizontal bars are the median value for the group NHP (n = 6 for G1, G2; n = 4 for G3, G4) and the vertical grey bars are the IQR.

Intracellular cytokine staining (ICS) analysis of non-Env and Env-specific blood T cells at week 6 (two weeks post the NYVAC-KC boost in G1 and G2) and week 22 (2 weeks post the final αLOX-1.Env gp140 administration) revealed robust Env-specific CD4^+^ T cell responses amplified from the weak responses primed by the NYVAC-KC administrations (G1 Nkc2Lp3Nkc and G2 Nkc2Lg3Nkc), or elicited in the naive NHPs (G3 Lp3Nkc and G4 Lg3Nkc) (Fig 7A). Env-specific CD8^+^ T cell responses were also detected in all groups. However, the Env-specific CD8^+^ T cell responses primed by NYVAC-KC were not amplified by αLOX-1.Env gp140 administration (Fig 7B).

**Fig 8:**
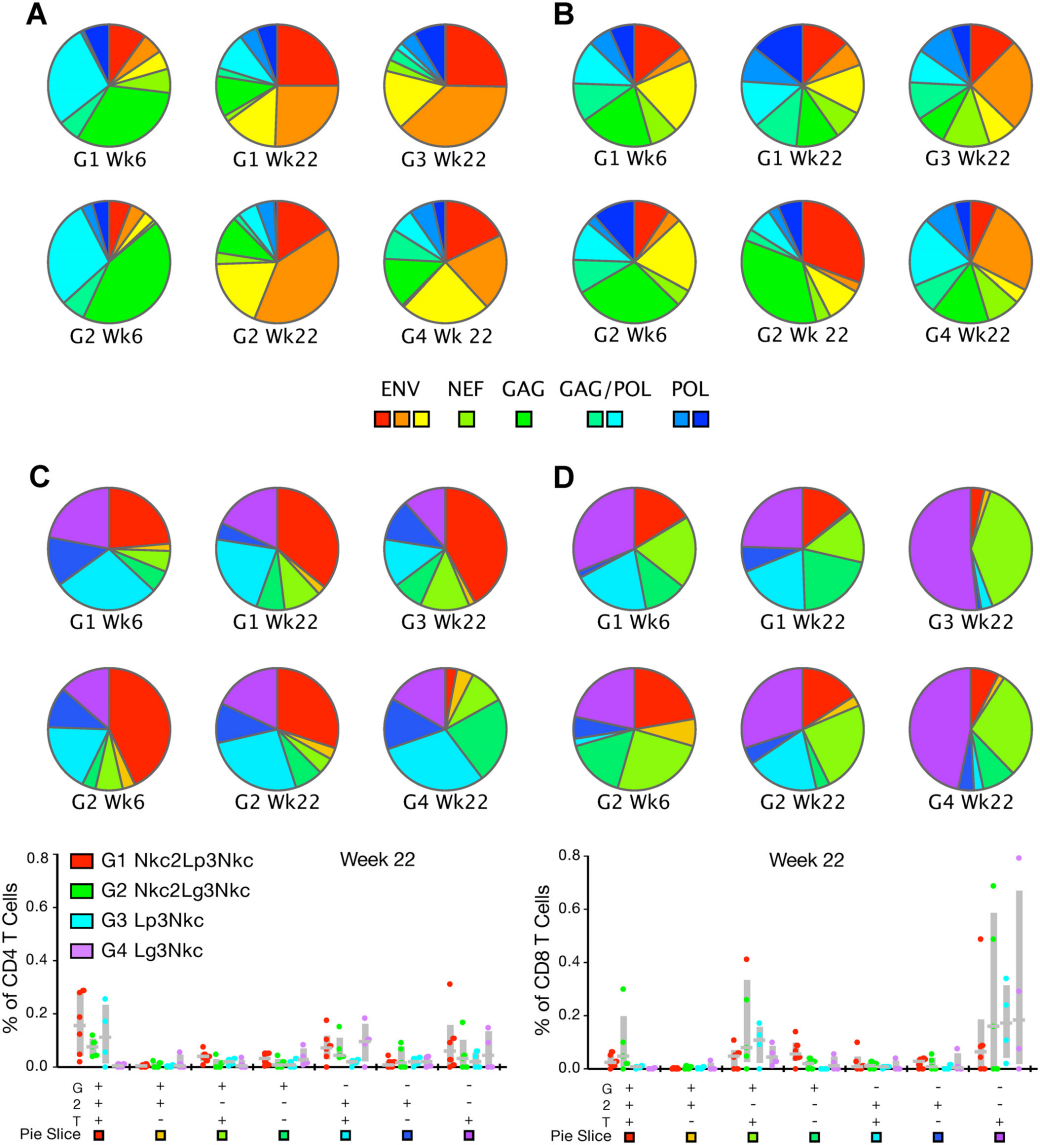
Analysis of HIV-specific T cell responses. The upper pie charts show the percentage of epitope specificities for (A) CD4^+^ T cell responses and (B) CD8^+^ T cell responses from PBMCs sampled at week 6 (2 weeks post NYVAC-KC administration, NHP groups G1 and G2 only) and week 22 (2 weeks post αLOX-1.Env gp140 administration) as defined by IFNγ^+^ T cells specific to each peptide pool. The lower pie charts show analysis of multifunctional HIV-specific T cell responses. ICS analysis of HIV-specific (C) CD4^+^ (left 6 pies) and (D) CD8^+^ (right 6 pies) T cells in PBMC samples taken at week 6 (2 weeks post NYVAC-KC administration, G1 Nkc2Lp3Nkc and G2 Nkc2Lg3Nkc only) and week 22 (2 weeks post αLOX-1.Env gp140 administration). The data show the breakdown of IFNγ^+^ (labeled as G), IL-2^+^ (labeled as 2) and TNFα^+^ (labeled as T) T cells specific to the combined HIV peptide pools (n = 6 NHP for G1, G2; n = 4 NHP for G3, G4). The lower linear presentation in (C) and (D) shows the same data as the pie charts, but includes data points for individual NHPs.

αLOX-1.Env gp140 vaccination expanded the proportion of Env-specific CD4^+^ T cells from all three Env regions sampled (Fig 8A), however, it had little effect on the specificity of the CD8^+^ T cell responses (Fig 8B). Antigen-specific T cell quality was appraised by intracellular production of IFNγ, TNFα, and IL-2. Quality was high (2–3 cytokines) for the CD4^+^ T cells in G1 Nkc2Lp3Nkc ands G2 Nkc2Lg3Nkc, both after the NYVAC-KC administrations and the αLOX-1.Env gp140 boosts (Fig 8C). For G3 Lp3Nkc and G4 Lg3Nkc, CD4^+^ T cell quality was high after the αLOX-1.Env gp140/ poly ICLC administrations, but low with αLOX-1.Env gp140/ GLA administrations. Quality was low to moderate (1–2 cytokines) for the HIV-specific CD8^+^ T cells in all groups (Fig 8D).

## Discussion

A milestone in the field of preventative HIV-1 vaccine was achieved with the reports of the RV144 trial, which evaluated a regimen consisting of a replication-defective vaccinia vector in combination with a recombinant gp120 protein in over 16,000 heterosexual men and women in Thailand [8,9]. This regimen demonstrated a statistically significant, albeit modest, reduction in HIV-1 acquisitions. While the first year vaccine efficacy (VE) approached 60%, this waned over time and overall VE over the 3.5 years of the trial was 31.2% [2].

Following these results, development of preventative vaccines against HIV-1 infection is focused on eliciting long-lasting humoral responses directed to the envelope protein. The desired properties of Env-specific antibodies likely include high affinity binding directed to relatively conserved regions of Env that are critical for cellular entry, and/or antibodies with ADCC properties to lyse infected cells.

While the RV144 study has shown that neutralizing antibodies were uncommon in protected individuals, essentially all RV144 vaccine recipients developed binding antibodies to gp120. Vaccinees with the highest levels of binding V1V2 antibodies were more likely to be protected than those with lower antibody levels [1,10,11,12]. Interestingly, the titer of these V1V2 antibodies waned over time in temporal association with the waning protective efficacy [12]. V1V2 antibodies were not broadly neutralizing but rather mediated ADCC and additional analyses showed the importance the IgG isotype, demonstrating that IgG3 antibodies had higher ADCC [12] while binding IgA Env antibodies were modestly associated with decreased vaccine efficacy [1,13]. However, evoking concomitant CD4^+^ T cell responses against Env or other HIV-1 proteins such as Gag [14] is also desired as helper CD4^+^ T cells can produce effector proteins to hinder infection and are essential for driving effective B cell responses, while cytotoxic CD8^+^ T cells can complement ADCC. This has been demonstrated in follow up analyses of the RV144 case-control data, where poly-functionality in CD4^+^ ENV-specific T cells was shown to be inversely correlated with infection risk [15].

It is likely that future success for HIV-1 vaccine development will rest on a prime-boost regimen of viral or DNA-based vectors encoding Env combined with other HIV-1 proteins for priming and Env protein as boost [16]. Questions addressing the exact nature of the Env sequence components enabling development of broadly neutralizing antibodies, the most potent adjuvants for Env protein administration, as well as the best regimen for the prime and the boost, are currently under investigation. Directing HIV-1-specific responses to the mucosal sites of primary infection is also essential for prevention of infection. Finally, in the RV144 study, the titers of anti V1V2 binding antibodies waned over time in association with the loss of protective efficacy suggesting that the vaccine strategy did not induce durable immune responses.

An unexplored vaccination approach is potential enhancement of humoral and cellular response directed to Env via targeting it to dendritic cells (DCs) with antibody vehicles specific for endocytic DC receptors. Here we present the first study in the NHP model of the efficacy of directing Env to LOX-1 expressed on various antigen-presenting immune cells, including DCs. We tested as an Env protein vaccine component αLOX-1.Env gp140 (clade C 96ZM651) adjuvanted with either poly ICLC or GLA in NHP in prime-boost combinations with NYVAC-KC, an attenuated vaccinia virus bearing HIV-1 Env gp140 (clade C 96ZM651) mixed with virus bearing Gag Pol and Nef. We chose to target Env to LOX-1 based on *in vitro* studies showing targeting to LOX-1 instructs DCs and B cells to promote mucosal plasmablast differentiation and elicits CD4^+^ T cell responses with a Th1 phenotype [5,6], as well as a pilot NHP study showing protective antibody responses to αLOX-1 vaccine bearing Influenza haemagglutinin [17].

Twice administered NYVAC-KC evoked low-level humoral immunity specific to Env and modest cellular responses specific to Env/Gag/Nef/Pol. A single boost with αLOX-1.Env gp140 combined with either poly ICLC or GLA was very effective in amplifying both anti-Env antibody and mixed CD4^+^/CD8^+^ T cell responses. Repeated αLOX-1.Env gp140 immunization favored the antibody response compared to the cellular response. The effectiveness of repeated αLOX-1.Env gp140 doses indicates that circulating antibodies directed either to Env or to the vehicle IgG4 component do not negate the action of the vaccine (also documented in reference [18]). In contrast, the NYVAC-KC boost following αLOX-1.Env gp140 administrations in primed animals had little discernable impact on humoral or cellular immunity directed to Env, possibly due to development of immunity to the vaccinia vector.

αLOX-1.Env gp140 administration in naïve NHP required two doses to elicit significant anti-Env gp140 humoral responses, with maximal observed responses after the third dose. Poly ICLC was moderately more effective than GLA for this antibody response, and helped raise modest antigen-specific serum IgA responses. Also, poly ICLC was clearly superior to GLA for evoking cellular immunity. In poly ICLC adjuvanted G3 Lp3Nkc NHPs, NYVAC-KC administration restored the cellular response to the peak level seen after the second αLOX-1.Env gp140 administration. These data indicate that αLOX-1.Env gp140 combined with poly ICLC is an efficient prime for NYVAC-KC boost.

Vaccine adjuvants based mainly on activators of innate immunity via TLR engagement are well studied, particularly in mice (reviewed in [19]), but direct comparisons of their efficacy in NHP versus man are limited. This is important since there are key differences in TLR distribution and action between mouse and man (see e.g., for TLR4 in [20]), but seeming concordance between `NHP and man (L. Su, personal communication). Poly IC, a synthetic analog of dsRNA recognized by TLR3, was previously shown to be effective for increasing antibody and CD4^+^ T cell responses against protein vaccines in NHP in both priming and viral boost settings [4,21,22]. GLA, a synthetic TLR4 agonist, which may have advantage in the setting of human intradermal injection [23] has previously proven to be effective in NHP for evoking humoral and CD4^+^ T cell immune responses against a malarial protein [24], and humoral immunity against Env in NHPs [25]. Our data for protein vaccine adjuvanted with poly ICLC are concordant with previous studies (above references and [5]), however the striking lack of T cell responses in naïve NHP administered with αLOX-1.Env gp140/GLA suggests negative interplay between antigen targeted to LOX-1-bearing APCs and direct or local indirect action of TLR4 stimulation. Mechanistic studies of antigen uptake and DC mobilization in the NHP dermal environment will help explore this phenomenon (see e.g., [18]).

The magnitude of vaccine-induced humoral and cellular responses is important, but their quality and longevity need to be sufficient to mount protective immunity. In this context, analysis of sera from peak response time points revealed similar data for all groups regarding the cross-reactivity and epitope breadth of Env-specific antibodies. The neutralizing antibody response was modest in magnitude and confined to sensitive clade C and AE viruses. In future studies mixed or sequential vaccinations with other HIV Env clade sequences should be investigated to circumvent this issue [25]. Importantly, CD4^+^ T cell quality at the peak response times was high, with breadth of epitopes across Env and ability to produce multiple effector cytokines. CD8^+^ T cell responses were elicited, but were somewhat less in magnitude and quality than CD4^+^ T cell responses. Similar to other observed vaccine-induced Env-specific humoral responses in NHP, serum IgG levels waned significantly over the 8-week observation period [26,27]. However, it is promising that in G3 Lp3Nkc and G4 Lg3Nkc NHPs the NYVAC-KC boost, akin to HIV-1 infection of a Env-protein vaccinated individual, rapidly restored high serum anti-Env IgG levels. Serum Env-specific IgA responses to αLOX-1.Env gp140, which is expected based on the role of LOX-1 in directing immunity to the mucosa [6], were observed but the expected protective potential of these antibodies is uncertain given the correlation of certain serum HIV-1-specific IgA responses with decreased vaccine efficacy in the RV144 trial [1].

An important issue not addressed in our present study is the relative efficacy of LOX-1-targeted Env versus non-targeted Env. However, a recent study [28] in Rhesus macaques used a similar protocol of two NYVAC-KC Env gp140 + GagPolNef administrations boosted by two administrations of gp120 protein at a similar molar dose (100 μg) to our study. Although the clade C gp120 sequences and adjuvant used were different, i.e., a mixture of TV1 gp120 with MF59 adjuvant and 1086 gp120 with alum (P), the immune monitoring assays were standardized across these two studies. In the closest matched groups of these two studies, a comparison between serum Env-specific antibody levels at the peak response times (G1 Nkc2Lp3Nkc, week 22 and [28] G1 Nkc2(NkcP)2, week 26) showed slightly weaker but very comparable (well within a mean difference of log_10_ magnitude) IgG responses against six tested Env antigens. Also, the HIV-1 neutralization titers at peak response times in these two groups were similar [28].

Further studies exploring lower dose ranges, longevity of responses, alternate routes of administration, and direct tests of viral challenge will be required to explore the possible benefits of αLOX-1.Env gp140 over non-targeted Env protein. Evaluation of Env-specific rectal and other mucosal IgA and IgG was not included in this study, but should be a component of future studies. The effect of targeting Env to other DC receptors is also unexplored, as is the possibility of increased breadth of neutralizing antibody development via targeting different Env sequences or forms, either concurrently or staged. Importantly, αLOX-1.Env gp140 administered with either GLA or poly ICLC adjuvant in both priming and boosting settings did not elicit adverse reactions in the NHPs. Demonstration of safety and efficacy in NHP is an essential step for justifying clinical development of αLOX-1.Env gp140.

## Materials and Methods

### Ethics statement

Twenty male Rhesus macaques ranging in age from 3 to 6 years and weighing at least 4 kg were procured from Harlan Laboratories and housed at the Advanced Biosciences Laboratories (ABL) animal facility in Rockville, Maryland. The ABL *in vivo* facility is USDA-registered and accredited by the American Association for the Accreditation of Laboratory Animal Care International (AAALAC). ABL’s veterinary practices comply with all policies of the "Guide for the Care and Use of Laboratory Animals," DHHS (NIH 85–23), Animal Welfare (DHHS-TN 73–2) the NIH Manual Issuance 4206 and 6000-3-4-58, "Responsibility for Care and Use of Animals CDC/NIH 4th edition”, “Biosafety in Microbiological and Biomedical Laboratories,” and Public Health Service Policy on Humane Care and Use of Laboratory Animals under a Category 1 assurance from OLAW. All procedures were carried out under Ketamine anesthesia by trained personnel under the supervision of veterinary staff and all efforts were made to ameliorate the welfare and to minimize animal suffering in accordance with the “Weatherall report for the use of non-human primates” recommendations. Other than for a seven-day post-inoculation follow up observation period, animals were pair-housed in adjoining primate cages allowing social interactions, under controlled conditions of humidity, temperature and light (12-hour light/12-hour dark cycles). Food and water were available *ad libitum*. Animals were monitored and fed standard laboratory rations twice daily. Trained personnel offered dietary supplements with fresh fruit and occasional treats at least once a day. The ABL primate environmental enrichment program aims to promote the psychological and physiological well being of non-human primates, including engagement in species-typical behavior. They are met through the following strategies:

i) Social enrichment through group housing; ii) sensory and cognitive enrichment through a novel food program, auditory and visual enrichment through presentation of approved music and visual stimuli plus distribution of foraging or novel toys/devices; iii) identification of and individualized treatment for psychological distress; iv) specialized enrichment for infants and juveniles and other special considerations; v) training to reduce or eliminate stress during human-animal interactions; and vi) program evaluation and documentation of daily assessments conducted by husbandry staff and quarterly behavioral assessments performed by the Program Veterinarian. Early endpoint criteria, as proposed by the project team and approved by the IACUC, were used to determine when animals should be humanely euthanized. The ABL veterinarian was authorized to determine whether animals met such criteria and if necessary, was tasked to stabilize any affected animals prior to consulting with the lead investigators. Criteria for determining the moribund state include: animal is unable to reach readily available food or water; animal shows no reaction to external stimulus; a serious fracture has occurred where the Veterinarian feels the pain cannot be controlled; normal bodily functions are prohibited by a tumor, ascites or infection; paralysis has occurred and recovery is unlikely; animal responds to severe pain/infection/inflammation by becoming anorexic and is unresponsive to medical treatment; presence of tissue necrosis and/or drainage which is not responsive to medical treatment; extreme body temperatures that cannot be controlled; signs of severe organ or system involvement; greater than 20% weight loss or chronic, uncontrollable diarrhea. The only additional endpoints outside of moribund sac and listed in the IACUC protocol were possible anaphylaxis which would have been treated by the Veterinarian. All animals in this study were euthanized at the protocol conclusion, following the current AVMA Guidance on Euthanasia, which was conducted by an overdose of intravenous sodium pentobarbitol. ABL’s Institutional Animal Care and Use Committee (IACUC) approved of the proposed study prior to the initiation of any *in vivo* work. The protocol number assigned by the IACUC/ethics committee that approved this study is AUP518.

### Vaccination protocols

The NYVAC-KC inoculum was provided in 0.2 ml aliquots as NYVAC-KC expressing Envgp140 (ZM96) and ZM96gag plus CN54polnef at 0.1 ml of each virus @1.2x10^8^pfu per vial. The immunogen mixture according to molar ratios was Env:GagPolNef = 1: 1. Prior to administration, the required number of vaccine vials containing the virus mixture were thawed at 37°C and put on ice immediately after thawing. 1 ml of 1xTBS was added to each vial and briefly vortexed. Sonication with a Bransonic 1210 unit was performed according to the following procedure: fill water bath of the sonicator with water and ice; sonicate sample 3 times for 10 seconds each time; after the sonication steps, vortex sample. For each animal, 1 ml of the vaccine preparation was drawn up from the vials by a 1 ml syringe and kept on ice until administration. Before immunization, the skin of the upper arm (deltoid) was shaved and cleaned with alcohol. Animals received the vaccine preparation via intramuscular injection of 1 ml of the vaccine preparation @2x108 pfu/ml total of 2 viruses in the deltoid. The Poly ICLC was provided in 1 ml vials at a concentration of 2 mg/ml. Vials (Hiltonol Lot: PJ215-1-10-01) were stored at 2–8°C. Administration was 1 mg (subcutaneous) in two injections of 250 μl in the center of each circular injection pattern (3–4 cm diameter) formed by the intradermal administrations of αDCIR HIV5pep or αCD40 HIV5pep vaccine components which were stored in 1 M Arginine + 100 mM Tris.HCl Buffer pH 6–8. Protein vaccine administrations were at a 200 μg dose, intradermal in a total of 8 injections of 250 μl each (2 ml total injection)—four injections will be performed in each side of the back placed in a circular pattern. To avoid toxicity of 1 M Arginine buffer, the concentrated protein will be diluted approximately 1:4 in PBS before use. Each animal received in the upper back (dorsal thoracic) a total of 8 intradermal injections of 250 μl each. Four injections were performed in each side of the back placed in a circular pattern of 3–4 cm of diameter. Skin was shaved before injection and cleaned with 70% alcohol solution. Intradermal injections were performed using an insulin syringe. The injection of Poly ICLC was performed after the I.D. injections of the proteins and in the middle of each circle with 500 μl injected S.C. This administration procedure was designed to promote drainage of antigen and adjuvant to the same lymph node site and at the recommendation of ABL’s head veterinarian. The veterinarian’s report concerning adverse events from vaccine formulation in the initial vaccinations is shown in S1 text.

### Production and quality assurance of αLOX-1.Env gp140

Vectors, cloning strategies, methods for deriving stably transfected CHO-S cells, their culturing and purification of secreted recombinant antibodies and fusion proteins have been described previously [29,30]. Specifically, αLOX-1.Env gp140 was derived from the parental anti-human LOX-1 15C4 recombinant human IgG4 mAb [5,6]; GenBank KM246787 and KM246788) via humanization of the mouse variable regions (Antitope, Ltd). Env gp140 sequence derived from the codon optimized HIV-1 96ZM651 synthetic construct (NIH AIDS reagent program, GenBank AY181197.1 residues 94–2064) was inserted at the IgG4 heavy chain C-terminal codon distal to a flexV1 flexible linker spacer [30] and proximal to 6 His codons. αLOX-1.Env gp140 was formulated at 0.5 mg/ml in 1 M Arginine 100 mM Tris.HCl pH 7.0 (neutralized protein A affinity elution buffer) and diluted 1:4 in PBS immediately before injection. In the initial I.D. vaccinations with αLOX-1.Env gp140 the 1:4 dilution in PBS was not done and grade 5 reactions to the vaccine was noted as pox-like lesions forming within 24–36 hours and then subsiding with the animals being otherwise normal. In subsequent vaccinations, with the 1:4 dilutions in 1xPBS, no similar reactions were observed (the full veterinary report is in S1 Text). LPS level was 130 pg/mg αLOX-1.Env gp140 protein. Specificity and relative binding affinity of αLOX-1.Env gp140 to human and Rhesus macaque LOX-1 ectodomains (encoded respectively by GenBank dbj|AB102861.1| residues 169–918 and ref|NM_001194668.1| residues 295–945) fused via the cohesin C-terminus (GenBank gb|CP000568.1|residues 3622666–3623172 with a Nhe I site linker) was confirmed by an adaption of multiplexed Luminex^®^ bead-based assay [17].

### Production of NYVAC-KC expressing ZM96gp140, ZM96Gag and CN54PolNef

NYVAC-KC was constructed as described earlier [31]. A negative selection cassette, PNR, was inserted into the TK locus of the virus by linear *in vivo* recombination. The cassette expresses the coumermycin dimerization domain of *Escherichia coli* gyrase B fused to the catalytic domain of mammalian dsRNA-dependent protein kinase, making the virus sensitive to coumermycin (described in [32]) following insertion; also in the cassette is a neomycin resistance gene. Plaques from the IVR were screened for neomycin resistance to identify viruses containing the cassette: NYVAC-KC-PNR. The candidate plaque with the greatest coumermycin sensitivity was then amplified for the next step. The plasmids plZAW1-gp140 (96ZM651) and plZAW1-Gag (96ZM651)-Pol-Nef (CN54) (described in [7], and provided by Ralf Wagner, University of Regensburg) were then used to insert the genes encoding gp140 and Gag-Pol-Nef into the TK locus of NYVAC-KC by *in vivo* recombination. Plaques from the IVR were screened in coumermycin-treated cells, first for blue color in the presence of β-galactosidase (lacZ gene in the plasmid, indicating a successful first recombination event to insert the plasmid) for two rounds, and then screened for loss of blue and resistance to coumermycin through two more rounds of plaque purifying (loss of the cassette equals gain of resistance to coumermycin, second recombination event removes plasmid features external to antigen coding, including the lacZ gene). IVRs were done in BSC-40 cells, stock virus preparation was done in BHK cells (both cell lines from ATCC), and stocks were 2X sucrose pad-purified. Correct antigen expression was verified by Western blot and Immunoplaque assay and genes were confirmed by sequencing. The two viruses, NYVAC-KC-gp140 and NYVAC-KC-GPN, were combined at a titer of 1x10^8^ pfu each virus per 0.2 ml per vial; contents of each vial were diluted just prior to inoculation in 1 ml 1X TBS for delivery of 2x10^8^ pfu per animal per 1 ml inoculation.

### Neutralization assay

Neutralization was measured as a function of reductions in luciferase (Luc) reporter gene expression after a single round of infection in TZM-bl cells as described [32]. TZM-bl cells were obtained from the NIH AIDS Research and Reference Reagent Program, contributed by John Kappes and Xiaoyun Wu. Briefly, test samples were diluted over a range of 1:20 to 1:43740 in cell culture medium and pre-incubated with virus (~150,000 relative light unit equivalents) for 1 hr at 37°C before addition of cells (10,000 cells in 100 μl of growth medium containing 75 μg/ml DEAE-dextran). Following incubation for 48 hr, cells were lysed and Luc activity determined using a micro titer plate luminometer and BriteLite Plus Reagent (Perkin Elmer). Neutralization titers are the sample dilution (for serum) or antibody concentration (for sCD4, purified IgG preparations and monoclonal antibodies) at which relative luminescence units (RLU) were reduced by 50% compared to RLU in virus control wells after subtraction of background RLU in cell control wells. Serum samples were heat-inactivated at 56°C for 1 hr prior to assay. Assay stocks of molecularly cloned Env-pseuotyped viruses were prepared by transfection in 293T cells and were titrated in TZM-bl cells as described [33].

### ELISA

Cohesin fusion proteins [30] were expressed in CHO-S cells from vectors containing the above Env gp140 sequence, or Env fragments (gp120 residues 94–1550; gp41 residues 1551–2064, inserted between the cohesin domain and 6 C-terminal His codons,). HIV Env-specific IgG antibody titers in serum from vaccinated animals were assessed at indicated time points post-immunization using ELISA. ELISA plates were coated with 2 μg/ml of the cohesin fusion proteins in 0.2 M sodium carbonate-bicarbonate buffer, pH 9.4. Serial dilutions of serum starting at 1:500 in TBS blocking solution (StartingBlock T20, Pierce) were incubated in the wells overnight at 4°C. After washing, plates were incubated with HRP-conjugated goat anti-human IgG (Jackson ImmunoResearch, West Grove, PA) in TBS blocking solution (Thermo Scientific, Rockford, IL) for 2 h at 37°C, then washed and developed with HRP substrate (TMB, Life Technologies), stopped with equal volume of 1N HCl and read at 450 nm. The log_10_ transformed and normalized areas under the curves (AUC) data were calculated for each animal at each time point using GraphPad Prism 6 software (GraphPad, La Jolla, CA). EC50 calculations were based on Log10 transformed and normalized data with non-linear regression curve fit using sigmoidal dose response with variable slope constraints.

### HIV-1 specific binding antibody assay

HIV-1 specific IgG antibodies to gp120/gp140 proteins and V1/V2 scaffolds were measured by an HIV-1 binding antibody multiplex assay [34]. All assays were run under GCLP compliant conditions, including tracking of positive controls by Levy-Jennings charts using 21CFR Part 11 compliant software. Positive controls included a HIVIG and CH58 mAb IgG titration. Negative controls included in every assay were blank, MulVgp70_His6 (empty gp70 scaffold) coupled beads, and HIV-1 negative sera. To control for antigen performance, we used the preset criteria that the positive control titer (HIVIG) included on each assay (and for assays with V1V2 antigens, CH58 mAb, [35]) had to be within +/- 3 standard deviations of the mean for each antigen (tracked with a Levy-Jennings plot with preset acceptance of titer (calculated with a four-parameter logistic equation, SigmaPlot, Systat Software). Antibody measurements were acquired on a Bio-Plex instrument (Bio-Rad, Hercules, CA) using 21CFR Part 11 compliant software and the readout is in MFI. The following antigens were examined: (Con S gp140 CF (a group M consensus envelope gp140; [36, 37], A1.con.env03 140 CF (clade A Consensus), B.con.env03 140 CF (clade B Consensus), C.con.env03 140 CF (clade C Consensus), ZM96 gp140 (clade C), JRFL gp140, 00MSA 4076 gp140, gp70 control, gp70_B.CaseA2 V1/V2 (a recombinant

clade B gp70 scaffold protein with the V1V2 variable region), C.1086V1-V2 Tags (a conformational V1-V2 antigen without the MuLV protein from transmitted clade C isolate that correlates with HIV-1 risk [10] provided by Drs. Liao and Haynes, Duke). Criteria for positive responses are defined below Statistical methods.

### Intracellular cytokine staining

Simulations were performed as described [38]. In short, cryopreserved PBMC were thawed and rested overnight in R10/RPMI 1640 (BioWhittaker, Walkersville, MD), 10% FBS, 2 mM L-glutamine, 100 U/ml penicillin G, 100 μg/ml streptomycin] with 50 U/ml Benzonase (Novagen, Madison, WI) in a 37°C/5% CO_2_ incubator. The following morning, cells were stimulated with peptide pools (2 μg/ml) in the presence of GogiPlug (10 μg/ml; BD Biosciences, San Jose, California) for 6 h. Negative controls received an equal concentration of DMSO instead of peptides. Subsequently, intracellular cytokine staining (ICS) was performed as described [39]. The following monoclonal antibodies were used: CD4-BV421 (clone OKT4; BioLegend), CD8-BV570 (clone RPA-T8; BioLegend), CD69-ECD (clone TP1.55.3; Beckman Coulter), CD3-Cy7APC (clone SP34.2; BD Biosciences), IFN-γ-APC (clone B27; BD Biosciences), IL-2-PE (clone MQ1-17H12; BD Biosciences), and TNF-FITC (clone Mab11; BD Biosciences). Aqua LIVE/DEAD kit (Invitrogen, Carlsbad, CA) was used to exclude dead cells. All antibodies were previously titrated to determine the optimal concentration. Samples were acquired on an LSR II flow cytometer and analyzed using FlowJo version 9.8 (Treestar, Inc., Ashland, OR).

### ADCC

ADCC activity against MW965.1 gp120 (provided by the Duke CAVD repository) coated CEM.NKR_CCR5_ (NIH AIDS Reagent Program, Division of AIDS, NIAID, NIH: CEM.NKR-CCR5 from Dr. Alexandra Trkola) target cells was measured using the ADCC-GranToxiLux (GTL) assay as previously described [40]. ADCC activity against HIV-1 infected cells was measured using the Luciferase ADCC assay as described [41]. For these assays, the CEM.NKR_CCR5_ target cells were infected with a replication-competent infectious molecular clone designed to encode the MW965.LucR.T2A.ecto/293T/17 (accession number U08455) *Env* gene in *cis* within an isogenic backbone that also expresses the *Renilla* luciferase reporter gene and preserves all viral open reading frames [42]. For both ADCC assay methodologies the effector cells were PBMC isolated from a HIV-1 seronegative human donor heterozygous for 158F/V polymorphic variants of Fcγ receptor 3A. The area under the curve was calculated to evaluate the impact of the vaccine regimen on the immune responses. For the GTL assay, the non-parametric area under the net activity percentage vs. dilution curve (AUC) was calculated based on log10 scale of dilution levels using trapezoid approach. The net activity percentage is calculated as difference between activity percentage of sample minus activity percentage of background, divided by % of target cell that are infected: ([Activity %]_sample_−[Activity %]_BKGD_)/[TARGET CELL INFECTED], where [TARGET CELL INFECTED] is set to 100 for coted cells. If the background activity is greater than the sample activity, the net activity percentage is set to zero for the sake of area calculation. For the Luc assay, the non-parametric area under the average percent loss Luciferase activity vs. dilution curve is calculated based on log10 scale of dilution levels using trapezoid approach. The average percent loss Luciferase activity is the average value for percent loss of Luciferase activity relative to background control over 2 wells: ([REDOUT VALUE]_BKG_-[READOUT VALUE])/[READOUT VALUE]_BKG -_, where [READOUT VALUE]_BKG_ is the average [READOUT VALUE] for wells containing Target and Effector cells, but no Ab, and [READOUT VALUE] is RLU for sample. If the average percent loss Luc activity is negative, the value is set to zero for the sake of area calculation.

### ELISPOT

Millipore 96 well filtration plates were pre-treated with 70% EtOH, washed 5x with 1x PBS and then coated with 5 μg/ml mouse-anti-human-IFNγ antibody (BD Pharmingen) over night at 4°C. After blocking with complete RPMI for 2 hours at 37°C, 2x10^5^ PBMCs (prepared as for ICS) were stimulated in triplicates with peptide pools at 1 μg/ml, or PHA (2.5 μg/ml) as positive control, while addition of medium only served as negative control. The peptides and peptide pools used are those described in [43] and are detailed in S4 Table. Plates were incubated at 37°C for 18–24 hours before washing with cold H_2_O twice and PBS/T for five times. Biotinylated anti-IFNγ-antibody (Mabtech) was added at 1 μg/ml for 1 hour at 37°C and, after washing, a 1: 2000 dilution of Avidin-HRP (Vector Laboratories) was added, again for 1 hour at 37°C. After final washing, stable DAB (Invitrogen) was added for 2 min, and then the reaction was stopped with water washing. After drying, the numbers of spots in each well were counted with an automated ELISPOT reader (CTL Immunospot).

### Statistical methods

Wilcoxon signed-rank tests are used to compare change of marker value at specific time points with baseline within each group. Comparison between groups at specific time points is made using the Wilcoxon rank sum test. Response rates are compared between groups using Fisher's exact test. Overall magnitude of ELISPOT response is compared between groups G1 and G2 by fitting a random effect model using log transformed data measured after W14. No adjustments are made for multiple comparisons, as these are exploratory analyses for which increased Type 1 error is tolerated for better sensitivity to detect effects. A p-value of less than or equal to 0.05 is considered statistically significant. Statistical analyses were done with R (version 3.1.2; The R foundation for Statistical Computing, Vienna, Austria). For the antigen-specific antibody measurements shown in Figs 5 and 6 several criteria are used to determine if data from an assay are acceptable and can be statistically analyzed. The blood draw rate must be within the allowable visit window as determined by the protocol. Secondly, if the blank bead negative control exceeds 5,000 MFI, the sample will be repeated. If the repeat value exceeds 5,000 MFI, the sample will be excluded from analysis due to high background. QC and standard curve titers must fall within +/- 3 standard deviations of the historical mean plotted on Levey Jennings charts. Sample and control replicates must also be within 20% CV. Samples from post-vaccination time points measured at 1:80 dilution level determined to have a positive binding antibody response by BAMA if they met three conditions: (1) the MFI minus Blank (MFI*) values at given dilution were greater than or equal to antigen-specific cutoff (based on the average +/- 3 standard deviation of 60 historical sero-negative plasma samples), (2) the MFI* values were greater than 3 times the baseline (day 0) MFI* values, and (3) the MFI values were greater than 3 times the baseline MFI values. If a background-subtracted Blank has negative MFI, the value was replaced by 0. For each antigen and visit, the magnitude of binding response among both responders and non-responders is compared between groups using the Wilcoxon rank sum test. Response rates are compared between groups using Fisher's exact test. No adjustments are made for multiple comparisons, as these are exploratory analyses for which increased Type 1 error is tolerated for better sensitivity to detect effects. A p-value of less than or equal to 0.05 is considered statistically significant.

## Supporting Information

S1 Fig Serum IgG titers against Env gp140 subdomains. (PDF)

S2 Fig ADCC-mediated antibody responses measured by GTL and Luciferase assays. (PDF)

S1 Table Reactivity of αLOX-1.Env gp140 versus anti-ENV mAbs. (PDF)

S2 Table Viral neutralization assay using TZM.bl cells using Tier 1 and Tier 2 HIV-1 Env pseudoviruses. (PDF)

S3 Table Viral neutralization assay using A3R5.7 cells using Env.IMC.LucR viruses made by transfection in 293T cells. (PDF)

S4 Table The peptides and peptide pools used for ELISPOT analysis. (XLSX)

S1 Text Veterinarian clinical case report. (PDF)
