# Supplementary material for: Targeting HIV-1 Env gp140 to LOX-1 Elicits Immune Responses in Rhesus Macaques
Source: PLoS One. 2016 Apr 14;11(4):e0153484. doi: 10.1371/journal.pone.0153484 (PMC4831750; doi:10.1371/journal.pone.0153484)
Supplement: S1 Fig — (PDF) [file pone.0153484.s001.pdf]

Supplemental Fig. 1

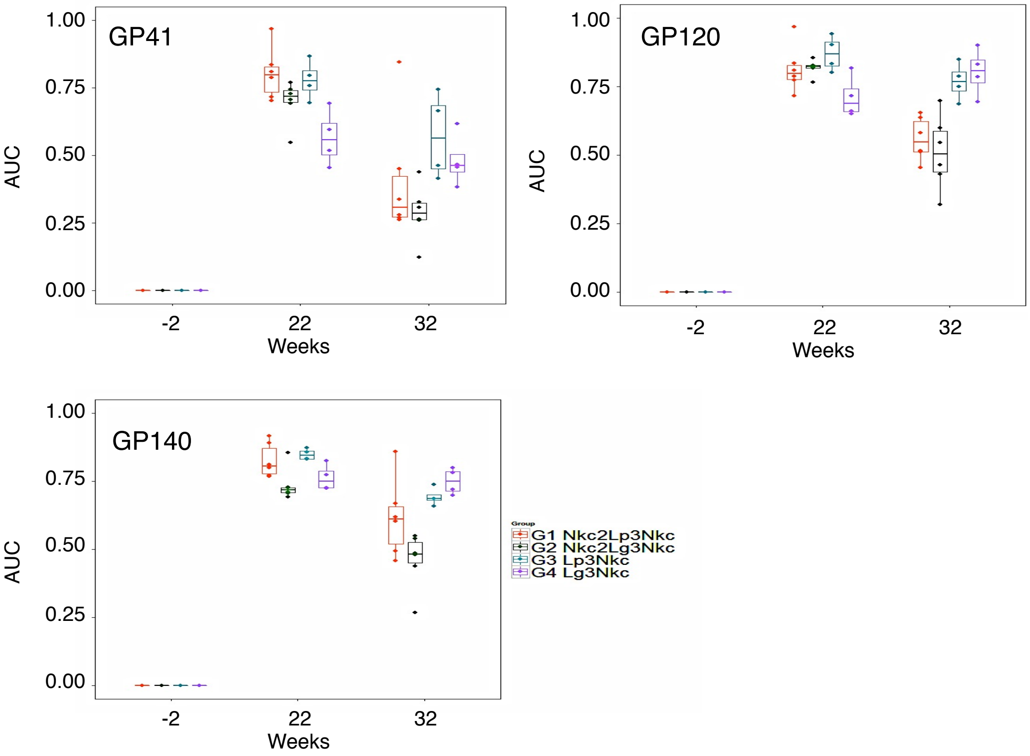

Serum IgG titers against Env gp140 subdomains. Results from individual solid phase ELISA against Env gp41 (A), gp120 (B), and gp140 (C) proteins from plasma samples from week -2 (baseline), week 22 (peak response, 2 weeks post αLOX-1.Env gp140 administrations), and week 32 (2 weeks post final NYVAC-KC boost). Data for individual NHP are shown as points. Each panel shows anti-Env IgG titers represented as area under the titration curve (AUC) of individual NHP responses (n=6 for G1, G2; n=4 for G3, G4). The raw data set was normalized across all groups setting the highest value as 1.00 and then GraphPad Prism software (La Jolla, CA.) was used to calculate AUC. The mid-line of the box denotes the median, and the ends of the box denote the 25th and 75th percentiles. The whiskers that extend from the top and bottom of the box extend to the most extreme data points that are no more than 1.5 times the interquartile range (i.e., the height of the box) or if no value meets this criterion, to the data extremes. Note that part of the data (weeks -2, 22, 32) in (C) is replicated independently in Fig. 2.
