# Supplementary material for: Targeting HIV-1 Env gp140 to LOX-1 Elicits Immune Responses in Rhesus Macaques
Source: PLoS One. 2016 Apr 14;11(4):e0153484. doi: 10.1371/journal.pone.0153484 (PMC4831750; doi:10.1371/journal.pone.0153484)
Supplement: S2 Fig — (PDF) [file pone.0153484.s002.pdf]

Supplemental Fig. 2

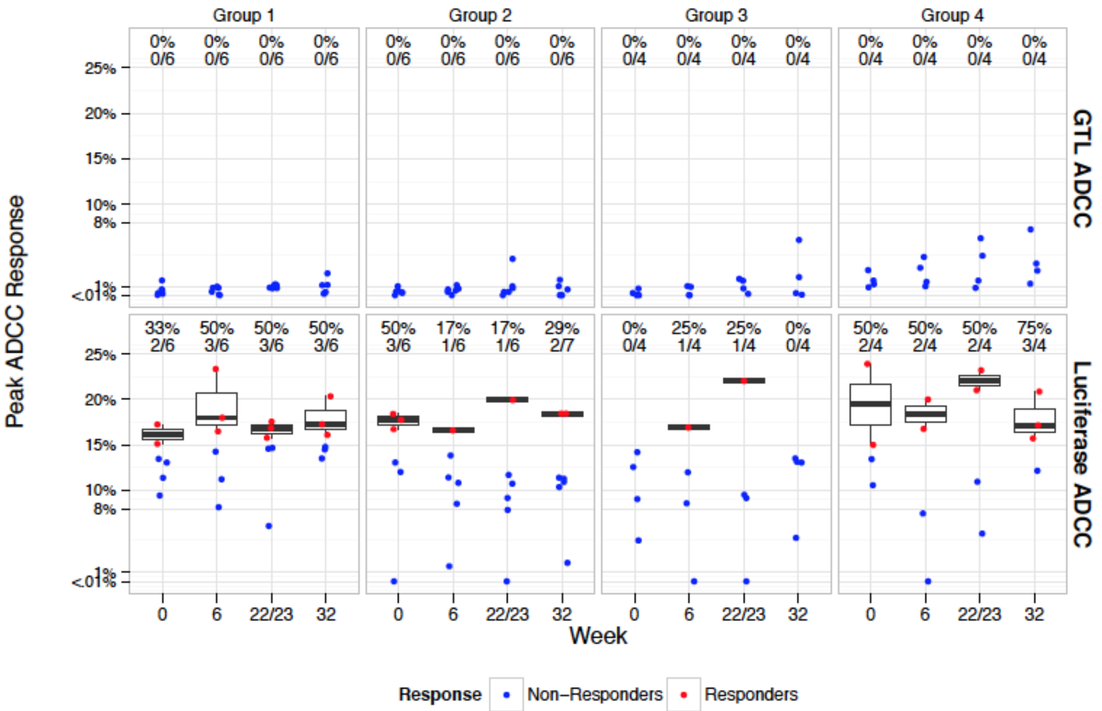

ADCC-mediated antibody responses measured by GTL and Luciferase assays. Response rates defined in the Materials and Methods are shown above the panels. Dots are data for individual animals and positive responses are shown in red, non-responses are shown in blue.
