# Supplementary material for: Targeting HIV-1 Env gp140 to LOX-1 Elicits Immune Responses in Rhesus Macaques
Source: PLoS One. 2016 Apr 14;11(4):e0153484. doi: 10.1371/journal.pone.0153484 (PMC4831750; doi:10.1371/journal.pone.0153484)
Supplement: S1 Table — (PDF) [file pone.0153484.s003.pdf]

Antibody	mAb Target	Binding to Env gp140 K_d / nM	Binding to αLOX-1. gp140 K_d / nM
HJ16	CD4 binding site	400 ± 38	51 ± 16
PGV04	CD4 binding site	n.d.	120 ± 32
b6	CD4 binding site	1.8 ± 0.3	39 ± 3.5
17b	CD4-induced	1.6 ± 1.8	36 ± 3.7
2G12	Glycan	280 ± 50	190 ± 4.8
HGN194	V3 loop	0.5 ± 0.4	72 ± 8.1

Supplemental Table 1.

Reactivity of αLOX-1.Env gp140 fusion protein versus anti-Env mAbs. Microscale thermopheresis binding analysis of αLOX-1.Env gp140 and matching trimeric Env gp140 protein was performed to measure the relative binding proficiency of a panel of mAbs (mean values of duplicate measurements are presented with calculated standard deviation).
