# Supplementary material for: Targeting HIV-1 Env gp140 to LOX-1 Elicits Immune Responses in Rhesus Macaques
Source: PLoS One. 2016 Apr 14;11(4):e0153484. doi: 10.1371/journal.pone.0153484 (PMC4831750; doi:10.1371/journal.pone.0153484)
Supplement: S2 Table — (PDF) [file pone.0153484.s004.pdf]

| | | Serum ID50 Titer in TZM.bl cells (1/x) | | | | | | | | | |
|---|---|---|---|---|---|---|---|---|---|---|---|
| | | Tier 1A (C) | Tier 1A (B) | Tier 1A (B) | Tier 1B (B) | Tier 1B (E) | | Tier 2 (C) | Tier 2 (C) | Tier 2 (C) | Neg. Control |
| **Group** | **Week** | **MW965.26** | **MN-3** | **SF162.LS** | **BaL.26** | **TH023.6** | | **TV1.21** | **Ce1086_B2** | **96ZM651** | **MuLV** |
| | | | | | | | | | | | |
| **G1** | -3 | <20 | <20 | <20 | <20 | <20 | | <20 | <20 | <20 | <20 |
| | 22 | **1,626** | <20 | <20 | <20 | **584** | | <20 | <20 | <20 | <20 |
| | 32 | **246** | <20 | <20 | <20 | **100** | | <20 | <20 | <20 | <20 |
| **G1** | -3 | <20 | <20 | <20 | <20 | <20 | | <20 | <20 | <20 | <20 |
| | 22 | **553** | <20 | <20 | <20 | **310** | | <20 | <20 | <20 | <20 |
| | 32 | **168** | <20 | <20 | <20 | **110** | | <20 | <20 | <20 | <20 |
| **G1** | -3 | <20 | <20 | <20 | <20 | <20 | | <20 | <20 | <20 | <20 |
| | 22 | **2,414** | <20 | <20 | <20 | **39** | | <20 | <20 | <20 | <20 |
| | 32 | **404** | <20 | <20 | <20 | **41** | | <20 | <20 | <20 | <20 |
| **G1** | -3 | <20 | <20 | <20 | <20 | <20 | | <20 | <20 | <20 | <20 |
| | 22 | **395** | <20 | <20 | <20 | **27** | | <20 | <20 | <20 | <20 |
| | 32 | **151** | <20 | <20 | <20 | **35** | | <20 | <20 | <20 | <20 |
| **G1** | -3 | <20 | <20 | <20 | <20 | <20 | | <20 | <20 | <20 | <20 |
| | 22 | **302** | <20 | <20 | <20 | **36** | | <20 | <20 | <20 | <20 |
| | 32 | **21** | <20 | <20 | <20 | <20 | | <20 | <20 | <20 | <20 |
| **G1** | -3 | <20 | <20 | <20 | <20 | <20 | | <20 | <20 | <20 | <20 |
| | 22 | **201** | <20 | <20 | <20 | **47** | | <20 | <20 | <20 | <20 |
| | 32 | **37** | <20 | <20 | <20 | <20 | | <20 | <20 | <20 | <20 |
| **G2** | -3 | <20 | <20 | <20 | <20 | **44** | | **25** | <20 | <20 | **25** |
| | 22 | **460** | <20 | <20 | <20 | <20 | | <20 | <20 | <20 | <20 |
| | 32 | **42** | <20 | <20 | <20 | <20 | | <20 | <20 | <20 | <20 |
| **G2** | -3 | <20 | <20 | <20 | <20 | <20 | | <20 | <20 | <20 | <20 |
| | 22 | **454** | <20 | <20 | <20 | **1,792** | | <20 | <20 | <20 | <20 |
| | 32 | **73** | <20 | <20 | <20 | **268** | | <20 | <20 | <20 | <20 |
| **G2** | -3 | <20 | <20 | <20 | <20 | <20 | | **25** | <20 | <20 | <20 |
| | 22 | **1,146** | <20 | <20 | <20 | **158** | | <20 | <20 | <20 | <20 |
| | 32 | **243** | <20 | <20 | <20 | **55** | | <20 | <20 | <20 | <20 |
| **G2** | -3 | <20 | <20 | <20 | <20 | <20 | | <20 | <20 | <20 | <20 |
| | 22 | **495** | <20 | <20 | <20 | <20 | | <20 | <20 | <20 | <20 |
| | 32 | **82** | <20 | <20 | <20 | <20 | | <20 | <20 | <20 | <20 |
| **G2** | -3 | <20 | <20 | <20 | <20 | <20 | | <20 | <20 | <20 | <20 |
| | 22 | **154** | <20 | <20 | <20 | <20 | | <20 | <20 | <20 | <20 |
| | 32 | **24** | <20 | <20 | <20 | <20 | | <20 | <20 | <20 | <20 |
| **G2** | -3 | <20 | <20 | <20 | <20 | <20 | | <20 | <20 | <20 | <20 |
| | 22 | **521** | <20 | <20 | <20 | <20 | | <20 | <20 | **27** | <20 |
| | 32 | **141** | <20 | <20 | <20 | <20 | | <20 | <20 | <20 | <20 |
| **G3** | -3 | <20 | <20 | <20 | <20 | <20 | | <20 | <20 | <20 | <20 |
| | 22 | **34** | <20 | <20 | <20 | <20 | | <20 | <20 | <20 | <20 |
| | 32 | **208** | <20 | <20 | <20 | <20 | | <20 | <20 | <20 | <20 |
| **G3** | -3 | <20 | <20 | <20 | <20 | <20 | | <20 | <20 | <20 | <20 |
| | 22 | **194** | <20 | <20 | <20 | **20** | | <20 | <20 | <20 | <20 |
| | 32 | **360** | <20 | <20 | <20 | **30** | | <20 | <20 | <20 | <20 |
| **G3** | -3 | <20 | <20 | <20 | <20 | <20 | | **21** | <20 | <20 | <20 |
| | 22 | **81** | <20 | <20 | <20 | <20 | | <20 | <20 | <20 | <20 |
| | 32 | **202** | **21** | <20 | <20 | <20 | | <20 | <20 | **25** | <20 |
| **G3** | -3 | <20 | <20 | <20 | <20 | <20 | | <20 | <20 | <20 | <20 |
| | 22 | **1,041** | <20 | <20 | <20 | <20 | | <20 | <20 | <20 | <20 |
| | 32 | **444** | <20 | <20 | <20 | <20 | | <20 | <20 | <20 | <20 |
| **G4** | -3 | <20 | <20 | <20 | <20 | <20 | | <20 | <20 | <20 | <20 |
| | 22 | **95** | <20 | <20 | <20 | <20 | | <20 | <20 | <20 | <20 |
| | 32 | **894** | **34** | <20 | <20 | **265** | | <20 | <20 | <20 | <20 |
| **G4** | -3 | <20 | <20 | <20 | <20 | <20 | | <20 | <20 | <20 | <20 |
| | 22 | **166** | <20 | <20 | <20 | <20 | | <20 | <20 | <20 | <20 |
| | 32 | **300** | <20 | <20 | <20 | <20 | | <20 | <20 | <20 | <20 |
| **G4** | -3 | **29** | <20 | <20 | <20 | **23** | | <20 | <20 | <20 | <20 |
| | 22 | **36** | <20 | <20 | <20 | **123** | | <20 | <20 | <20 | <20 |
| | 32 | **97** | <20 | <20 | <20 | **158** | | <20 | <20 | <20 | <20 |
| **G4** | -3 | <20 | <20 | <20 | <20 | <20 | | <20 | <20 | <20 | <20 |
| | 22 | **115** | <20 | <20 | <20 | **22** | | <20 | <20 | <20 | <20 |
| | 32 | **403** | **60** | <20 | <20 | **56** | | <20 | <20 | <20 | <20 |

**Supplemental Table 2. Viral neutralization assay using TZM.bl cells using Tier 1 and Tier 2 HIV-1 Env pseudoviruses.** Samples were tested as primary serum1:20 titrated 3-fold 7x (duplicate wells). Values are the serum dilution at which relative luminescence units (RLUs) were reduced 50% compared to virus control wells (no test sample). Values in bold type scored positive for neutralization based on the criterion of > 3X the observed background in the pre-bleed.
