# Supplementary material for: Targeting HIV-1 Env gp140 to LOX-1 Elicits Immune Responses in Rhesus Macaques
Source: PLoS One. 2016 Apr 14;11(4):e0153484. doi: 10.1371/journal.pone.0153484 (PMC4831750; doi:10.1371/journal.pone.0153484)
Supplement: S3 Table — (PDF) [file pone.0153484.s005.pdf]

Group	Week	Curve-based IC50 in A3R5.7 Cells[1,2]				
		TV1.21.LucR.T2A.ecto	Ce1086_B2.LucR.T2A.ecto	Du151.2.LucR.T2A.ecto	Ce1176_A3.LucR.T2A.ecto	Ce2010_F5.LucR.T2A.ecto
		ID#5069	ID#4577	ID#5352	ID#5330	ID#5479
**G1**	-3	<20	<20	<20	<20	21
	22	**47**	<20	<20	**21**	29
	32	**23**	<20	<20	<20	23
**G1**	-3	<20	<20	<20	<20	27
	22	**63**	**42**	<20	**21**	59
	32	**25**	**27**	<20	<20	<20
**G1**	-3	<20	<20	<20	<20	<20
	22	**57**	<20	<20	<20	<20
	32	**38**	**24**	<20	<20	<20
**G1**	-3	28	<20	<20	24	<20
	22	35	<20	<20	<20	<20
	32	31	**21**	<20	<20	<20
**G1**	-3	26	<20	<20	<20	<20
	22	59	<20	<20	**24**	**33**
	32	<20	<20	<20	<20	<20
**G1**	-3	<20	<20	<20	<20	<20
	22	**36**	<20	<20	<20	**22**
	32	**20**	<20	<20	<20	**22**
**G2**	-3	<20	<20	<20	<20	<20
	22	**55**	**57**	<20	**28**	**38**
	32	<20	<20	<20	<20	**22**
**G2**	-3	<20	<20	<20	<20	23
	22	**39**	<20	<20	<20	<20
	32	<20	<20	<20	<20	20
**G2**	-3	<20	<20	<20	<20	29
	22	**48**	<20	<20	<20	21
	32	<20	<20	<20	<20	<20
**G2**	-3	<20	<20	<20	<20	<20
	22	**26**	<20	<20	<20	**22**
	32	<20	<20	<20	<20	**27**
**G2**	-3	<20	<20	<20	<20	<20
	22	**25**	<20	<20	<20	**28**
	32	**28**	**21**	<20	<20	**26**
**G2**	-3	32	<20	<20	23	23
	22	**124**	**62**	**22**	37	44
	32	34	<20	<20	<20	<20
**G3**	-3	<20	<20	<20	<20	<20
	22	<20	<20	<20	<20	<20
	32	<20	<20	<20	<20	<20
**G3**	-3	24	<20	<20	<20	<20
	22	39	<20	<20	**26**	**20**
	32	50	<20	<20	<20	**29**
**G3**	-3	24	<20	<20	<20	<20
	22	51	<20	<20	**30**	**34**
	32	51	<20	<20	<20	<20
**G3**	-3	24	<20	23	31	22
	22	**82**	<20	<20	23	25
	32	58	<20	<20	<20	<20
**G4**	-3	25	<20	<20	<20	<20
	22	26	<20	<20	<20	**29**
	32	49	**22**	<20	<20	**23**
**G4**	-3	<20	<20	<20	<20	<20
	22	**28**	<20	<20	<20	<20
	32	**32**	<20	<20	<20	<20
**G4**	-3	43	<20	<20	<20	<20
	22	<20	**21**	<20	<20	<20
	32	23	<20	<20	<20	<20
**G4**	-3	55	<20	23	51	<20
	22	67	<20	21	39	<20
	32	107	**66**	34	35	**24**

Supplemental Table 3. Viral neutralization assay using A3R5.7 cells using Env.IMC.LucR viruses made by transfection in 293T cells. [1]Values are the serum dilution at which relative luminescence units (RLUs) were reduced 50% compared to virus control wells (no test sample). [2]Values in bold type scored positive for neutralization based on the criterion of > 3X the observed background in the pre-bleed.
