# Supplementary material for: Targeting HIV-1 Env gp140 to LOX-1 Elicits Immune Responses in Rhesus Macaques
Source: PLoS One. 2016 Apr 14;11(4):e0153484. doi: 10.1371/journal.pone.0153484 (PMC4831750; doi:10.1371/journal.pone.0153484)
Supplement: S1 Text — (PDF) [file pone.0153484.s007.pdf]

Supporting Information

S1 Text. Veterinarian clinical case report

Overview of Immunizations:

3.20.12- NYVAC-KC or Sham immunization

4.17.12- NYVAC-KC or Sham immunization

6.11.12 to 6.13.12- αLOX-1/Poly ICLC or αLOX-1/GLA immunization

7.10.12- αLOX-1/Poly ICLC or αLOX-1/GLA immunization

8.7.12- αLOX-1/Poly ICLC or αLOX-1/GLA immunization

10.16.12- NYVAC-KC immunization

Immunization Observations:

3.20.12- NYVAC-KC or Sham immunization: No notable observations.

4.17.12- NYVAC-KC or Sham immunization: No notable observations.

6.11.12 to 6.13.12- αLOX-1/Poly ICLC or αLOX-1/GLA immunization:

All animals in all groups developed pox-like lesions within 24-36 hours of the immunization at the 5 intra-dermal αLOX-1 gp140 vaccination sites due to a reaction to the 1M L-arginine/Tris storage buffer. These lesions were raised, red, indurated, with oozing crusts and heavy scabs, but no observable pruritis. The diameter of the lesions ranged from 5 to 8 mm and were considered grade 4/5 (see table below) except for one animal (R347 in group 2) that only developed grade 3 lesions. Lesions appeared to peak in severity at day 4-5 post vaccination and resolved in approximately 4 weeks, just prior to the next scheduled immunization. There were no obvious differences between study treatment groupings and no inflammation was seen at the adjuvant site in any group, delivered by SC injection. Animals did not become systemically ill, their appetites, behavior and comfort levels were all normal during this time.

Table: Lesion Scoring

	Mild (verging on normal) (Grade 1)	Mild (Grade 2)	Moderate (Grade 3)	Severe (Grade 4)	Potentially Life Threatening (Grade 5)
Erythema	Pale pink	Pink	Red	Dark Red	Necrosis
Induration	<2 mm	2-3 mm	3.1-4 mm	4.1-5 mm	>5 mm and necrosis

Scoring based on previous experience grading small pox and TB vaccine lesions. Induration is described as a palpably hard, raised area. Erythema is described as pink to red coloration of skin.

The root cause of the lesions was identified through a pilot experiment conducted on unrelated cynomolgus macaques using ID injections of 3 different lots of αLOXx-1 gp140 fusion protein plus the 1M L-arginine/Tris storage buffer alone. Each of the test conditions (i.e., fusion protein alone or buffer alone) were administered in duplicate at three concentrations (undiluted, 1:2 and 1:5) in 1x PBS. The results of the pilot experiment strongly suggested that the dermal reaction witnessed after the first round of ID injections was due to the administration of undiluted L-arginine/Tris storage buffer and not the αLOX-1 gp140 antigen itself. This pilot experiment further suggested that dilution of the storage buffer with 1x PBS (in the range of 1:2 to 1:5) would yield weak/undetectable reactions at the ID injection sites. As a result, the IACUC protocol was revised to account for a 1:4 dilution of the αLOX-1 gp140 vaccine prior to administration and for a change in the location of future αLOX-1 gp140 immunizations to the dorsal thoracic area, in order to promote drainage to the same lymph node site.

7.10.12- αLOX-1/Poly ICLC or αLOX-1/GLA immunization:

With the 1:4 dilution of the αLOX-1 gp140 fusion protein/storage buffer preparation and injection amendments made to the IACUC protocol, veterinary staff did not see the same severe level of reactivity observed from the June immunizations. Mild reactions were noted in only 3 out of 20 animals, including slight redness and minimal scabbing. One monkey, R349, had a grade 3 reaction with small scabs and mild erythema while other monkeys did not exceed a mild, grade 1 reaction. All reactions advanced into the resolution phase within a few days.

8.7.12- αLOX-1/Poly ICLC or Lox-1/GLA immunization:

Very mild reactions were seen during this post-immunization observation period. Animal R349 had a grade 3 reaction at the ID injection sites and the Veterinarian was unable to use the same location for inoculation on this animal because the lesions had not quite completely resolved from the July 10th immunization. Animal R347 exhibited a grade 2 reaction, but all reactions were in the resolution phase about a week after administration. With the exception of R349, the Veterinarian believed that the reactions were the result of the large administration volume going intra-dermally and causing some tissue trauma.
